# Extensive stage-regulation of translation revealed by ribosome profiling of *Trypanosoma brucei*

**DOI:** 10.1186/1471-2164-15-911

**Published:** 2014-10-20

**Authors:** Bryan C Jensen, Gowthaman Ramasamy, Elton J R Vasconcelos, Nicholas T Ingolia, Peter J Myler, Marilyn Parsons

**Affiliations:** Seattle Biomedical Research Institute, 307 Westlake Ave N, Seattle, WA 98109-5219 USA; Department of Molecular and Cell Biology, University of California, Berkeley, CA 94720-3202 USA; Department of Global Health, University of Washington, Harris Hydraulics Building, 1705 NE Pacific St #310E, Box 357965, Seattle, WA 98195 USA; Department of Biomedical Informatics and Medical Education, University of Washington, Seattle, WA 98195 USA

**Keywords:** Ribosomal proteins, Ribosome profiling, Stage-regulation, Trypanosome, Translation

## Abstract

**Background:**

*Trypanosoma brucei* subspecies infect humans and animals in sub-Saharan Africa. This early diverging eukaryote shows many novel features in basic biological processes, including the use of polycistronic transcription to generate all protein-coding mRNAs. Therefore we hypothesized that translational control provides a means to tune gene expression during parasite development in mammalian and fly hosts.

**Results:**

We used ribosome profiling to examine genome-wide protein synthesis in animal-derived slender bloodstream forms and cultured procyclic (insect midgut) forms. About one-third of all CDSs showed statistically significant regulation of protein production between the two stages. Of these, more than two-thirds showed a change in translation efficiency, but few appeared to be controlled by this alone. Ribosomal proteins were translated poorly, especially in animal-derived parasites. A disproportionate number of metabolic enzymes were up-regulated at the mRNA level in procyclic forms, as were variant surface glycoproteins in bloodstream forms. Comparison with cultured bloodstream forms from another strain revealed stage-specific changes in gene expression that transcend strain and growth conditions. Genes with upstream ORFs had lower mean translation efficiency, but no evidence was found for involvement of uORFs in stage-regulation.

**Conclusions:**

Ribosome profiling revealed that differences in the production of specific proteins in *T. brucei* bloodstream and procyclic forms are more extensive than predicted by analysis of mRNA abundance. While *in vivo* and *in vitro* derived bloodstream forms from different strains are more similar to one another than to procyclic forms, they showed many differences at both the mRNA and protein production level.

**Electronic supplementary material:**

The online version of this article (doi:10.1186/1471-2164-15-911) contains supplementary material, which is available to authorized users.

## Background

Most organisms exhibit robust gene regulation at the level of transcription. Among the exceptions to this rule are the trypanosomatid parasites, including *Trypanosoma brucei*. Nonetheless, as *T. brucei* transits its life cycle through the mammalian and insect hosts, large changes in protein expression occur [1–3]. Whilst previous microarray and RNA-seq studies [4–12] have shown that a moderate number of transcripts are developmentally regulated, primarily as a result of differential mRNA stability [13–15], much less is known about the role of translational regulation. A limited number of individual genes have been shown to be developmentally regulated at the level of translation [15–18] and numerous examples of discrepancies between stage-specific changes in mRNA and protein level exist. A recent study has noted some changes in the association of mRNAs with polysomes in developing mammalian bloodstream forms (BF) [19] and initial work suggests that the changes in translation efficiency occur between cultured BF and cultured insect stages [20]. The work presented here aimed to define the role of translational control in modulating differences in gene expression during parasite development.

*Trypanosoma brucei* spp. are the causative agents of lethal human African trypanosomiasis (African sleeping sickness) and nagana, a wasting disease in cattle. The presence of *T. brucei* and related African trypanosomes in sub-Saharan Africa has had a major impact on development, affecting humans directly as well as indirectly through impact on livestock. African trypanosomes share molecular mechanisms of gene regulation with the agents of Chagas’ disease (*Trypanosoma cruzi*) and leishmaniasis (*Leishmania* spp.). The most striking feature of nuclear gene expression in these organisms is the organization of genes into long polycistronic clusters, such that individual genes lack promoters [21–23]. The polycistronic mRNAs are processed into individual transcripts by *trans*-splicing of a common capped mini-exon sequence (the spliced leader, SL) to the downstream coding sequence (CDS) and concomitant polyadenylation of the upstream gene [24]. Thus all mRNAs bear the same sequence at their 5′ terminus, upstream of the gene-specific untranslated region. Despite the ubiquity of *trans*-splicing, *cis*-splicing is extremely rare, with only two known examples in *T. brucei*. Interestingly, mRNAs derived from the same polycistronic cluster are not generally expressed to similar levels, nor do they tend to show the same patterns of developmental regulation. These differences in mRNA abundance are thought to be mediated post-transcriptionally, in large part by differential stability resulting from interactions with RNA binding proteins [13, 25].

The extent of gene regulation at the mRNA level is a major contributor to differential protein expression in most species. However, additional levels of regulation are known to yield different levels of expression of various proteins under a given condition as well as modulating how those levels change upon perturbation. For example, a recent study that dissected the contributions of level of transcription, mRNA turnover, translation, and protein degradation demonstrated that translational efficiency was the largest contributor to predicting protein abundance across genes [26]. Factors that contribute to translational efficiency include gene-specific features, such as the context of the start codon, the presence of upstream open reading frames (uORFs), the length and sequence composition of the 5′ UTR, and the presence of protein binding sites in the untranslated regions (UTRs) [27–29]. Changes in the cellular milieu can also affect translation by modulating abundance or modification of translation factors (*e.g.*, phosphorylation of eIF2α), altering the abundance of different RNA binding proteins or microRNAs, perturbing protein folding and changing polyadenylation [29, 30]. Other studies have shown that translational controls play a prominent role in oncogenesis of mammalian cells [31], induction of the unfolded protein response in *Toxoplasma gondii* [32], exposure to light in *Arabidopsis* [33], and during development in *Plasmodium* [34, 35], to name a few. In trypanosomatids, given the lack of transcriptional controls, we hypothesized that translational regulation would play a prominent role in parasite development.

To directly test this hypothesis, we made use of the recently developed technique of ribosome profiling [36, 37], which quantitatively interrogates the positions of all ribosomes on their mRNA templates, thus providing a comprehensive picture of cellular translation. Our results show extensive changes in gene-level protein production between *in vivo*-derived slender BF (slBF) and insect midgut stages (procyclic cultured forms, PCF), greater in extent and magnitude than changes in mRNA abundance. Of the 8398 intact genes studied, thousands of genes show changes in protein synthesis mediated by both mRNA abundance and translational efficiency, but less than 200 genes appear to be regulated by changes in translation efficiency. Comparison with cultured BF (cBF) from another strain allowed the further definition of changes in protein production associated with growth conditions and strain variation.

## Results and discussion

**Figure 1:**
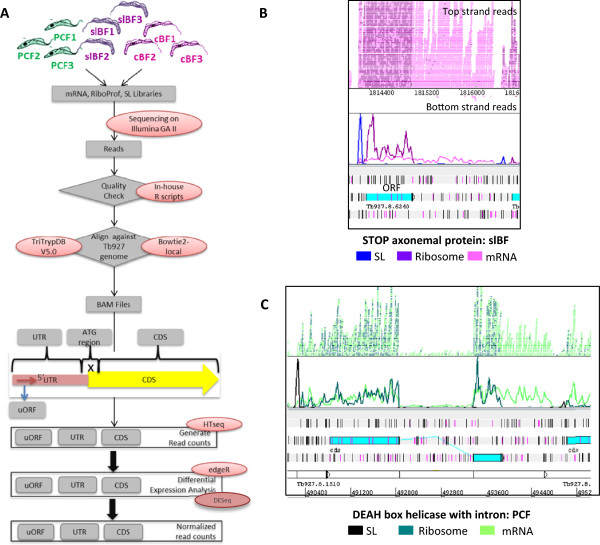
**The ribosome profiling system. A)** Diagram of work flow. **B)** Visualization of the sequence mapping onto the genome in Artemis. This image shows the spliced leader, ribosome footprint and mRNA reads mapping to the region of the STOP axonemal protein gene in slBF. Reads are color-coded as shown below the image; data for expression in slBF are shown in purple-pink throughout the manuscript. Here and elsewhere, start codons are shown in pink in the three reading frames while stop codons are black. The numbers under the stacked reads correspond to the coordinates in the chromosome and the GeneID for TriTrypDB is shown. **C)** SL, ribosome profiling, and mRNA reads mapping to the region of a DEAH box helicase gene in PCF. This gene is one of only two genes in the *T. brucei* genome that has an intron. Note the lack of ribosome profiling reads in the intron even though a low level of mRNA is present. Data for expression in PCF are shown in blue-green colors throughout.

The total protein synthetic activity devoted to a given gene is determined by both its mRNA abundance and the efficiency with which its mRNA is translated. To assess the extent of translational regulation during *T. brucei* development we pursued a genome-wide ribosome profiling approach [36]. Our primary focus was on two rapidly proliferating life cycle stages (PCF and slBF) that are readily available for the pleiomorphic *T. brucei* strain 927, which has retained the ability to differentiate and also has the most complete genome sequence available. We also examined cultured BF (cBF) from another often-used strain, *T. brucei* 427 (see Additional file 1: Table S1 for sample description). Figure 1A shows an overview of the workflow, with libraries being prepared and sequenced from three biological replicates of each condition. This approach relies on the ability of the translating ribosome to protect a footprint of ~28 nt from RNase digestion (see Additional file 2: Figure S1). Ribosome-protected fragments are purified and used to generate libraries for high throughput sequencing. The read counts from these libraries reflect the extent of translation of each gene, allowing quantitative measurement of gene expression between samples. Moreover, comparison with the read counts from fragmented poly(A) + mRNA libraries prepared using the same biological samples reveals the relative contributions of changes in mRNA abundance and translational efficiency to regulation of protein production. We also constructed libraries that specifically captured the 5′ ends of the mRNAs using SL RNA-seq (see Methods), assisting in refinement of the annotated CDSs.

Reads were mapped to the *T. brucei* 927 genome and assigned to individual genes (as described in Methods), yielding 6-15 million uniquely mapping reads per biological sample (see Additional file 1: Table S2 for statistics). Additional file 3 provides gene-level read count data for all 9141 annotated CDSs, newly identified CDSs, and pseudogenes. The ribosome footprints showed the characteristic 3 nucleotide periodicity, being enriched for reads starting at the first nucleotide of each codon (Additional file 2: Figure S1c), while mRNAs reads were relatively evenly distributed across the nucleotide positions. As expected, mRNA reads extend from the site of the SL through the CDS and terminate at the most 3′ polyA site, usually ~100 nt upstream of the predominant SL site of the downstream gene. In contrast, ribosome footprint reads span the CDS from 12 nt prior to start codon to 9 nt past the stop codon and are absent from the 3′ untranslated region (UTR) (see Additional file 1: Table S3 and Additional file 2: Figure S4). Ribosome release scores (RRSs, also referred to as the disengagement score), a metric of translation that compares the density of ribosome footprints on the CDS to that in the 3′ UTR, using mRNA as a control, were calculated for each intact CDS (except the ~8% for which read counts were very low, see Methods) [38, 39]. Of those genes for which scores could be calculated, 87% had scores >10 and 60% had scores >50, indicating most mRNAs had considerable enrichment for ribosome footprints in the CDS (see Additional file 3). Technical replicates showed high reproducibility in read counts for both ribosome footprints and mRNA, whereas biological replicates showed more variation (Additional file 2: Figure S2A). Nonetheless, correlation coefficients between gene-level read counts for biological replicates were high for both ribosome footprints (r^2^ = 0.86-0.94) and mRNA (r^2^ = 0.74-0.94) (Additional file 2: Figure S3). Both ribosome footprint and mRNA reads were highly strand-specific (see Figure 1B), but the distributions of edgeR [40] normalized read counts per gene were noticeably different between ribosome footprints and mRNA (Additional file 2: Figure S2B), with the ribosome footprint read counts showing a broader spread than the mRNA reads. Only two *T. brucei* genes undergo both *cis* and *trans*-splicing. For these genes, ribosome footprint reads are abundant in the exons, but absent in the intron, whereas there are readily detectable mRNA reads in the latter (see Figure 1C).

To assess whether RNA binding proteins might protect the portion of the mRNA with which they interact to yield similarly sized fragments as ribosome protection (and that RNA-protein complex would sediment under the conditions used to pellet the ribosome), we examined several transcripts with 3′ UTRs known to bind specific proteins. No significant ribosome footprint peaks were observed within the 3′ UTRs of the *GPEET2*, *ZC3H11* and *PGKB* mRNAs (see Additional file 2: Figure S5), despite extensive evidence that they bind multiple different proteins [41–43]. As seen in Figure 1B, there was often a ribosome footprint peak close to the CDS start codon (see Additional file 2: Figure S4), possibly due to an artifact of cycloheximide treatment [36], which blocks elongation but not initiation. For this reason, the first 45 nt of the CDS were not included in the gene-level read counts (see Methods). Although *T. brucei* 5′ UTRs are generally short (the median from our SL data being 87 nt, not including the SL itself) many of the longer 5′ UTRs clearly show ribosome footprints that are distinct from the peak at the start codon (Figure 1C). The median ribosome footprint read density in the 5′ UTRs correlated with those of the corresponding CDSs (R^2^ = 0.43-0.62, depending on the condition), but was generally lower (Additional file 1: Table S3). Recent work has demonstrated that ribosome footprints on noncoding RNAs and noncoding regions of mRNAs (such as the 5′ UTR) can be discriminated from translation of functional protein-coding genes because the profile does not terminate at stop codons, likely due to the presence of weak translation in multiple overlapping reading frames [38, 39]. For most genes in our study, these 5′ UTR footprints were not associated with any ORFs starting with an ATG, and they continued through the 5′ UTR irrespective of the presence of stop codons; hence in most cases they do not represent specifically translated upstream ORFs (uORFs). These protected fragments may represent assembled 80S ribosomes, as suggested from similar observations in yeast and mammalian cells [44] or possibly protection by the scanning complex.

### General aspects of the translational landscape

**Figure 2:**
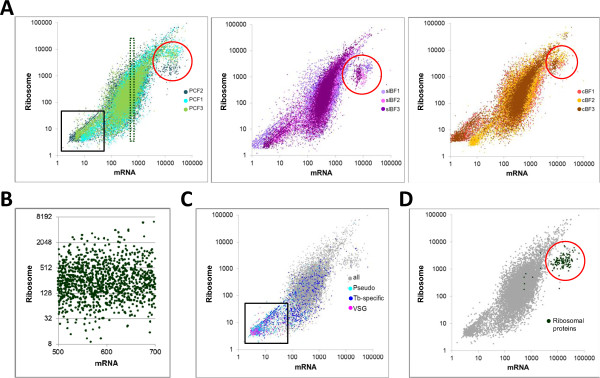
**Overview of the translational landscape. A)** Ribosome footprint and mRNA edgeR-normalized RPK for all genes, including pseudogenes, are shown. Each panel includes all biological replicates for a given stage, which are shown in different shades. The box outlines the genes with <50 RPK, the dotted box is enlarged in panel B; and the circle marks a set of genes with high mRNA read counts but relatively lower ribosome read counts that is referred to in the text. **B)** Illustration of large differences in ribosome association with mRNAs expressed to similar levels in PCF sample 2. Note that the x-axis is linear and the y-axis is log_2_. **C)** Expression levels of pseudogenes, VSG genes and *T. brucei* specific genes in PCF. The boxed area (<50 RPK) is comprised mostly of pseudogenes (cyan dots) and VSG genes (pink dots) and a subset of the *T. brucei* specific genes (blue dots). **D)** The cluster of genes with reduced translation efficiency corresponds to structural components of the cytosolic ribosome (green dots).

Ribosome profiling provides a comprehensive overview of the genes to which cells devote the most translational resources, which reflects a far greater biosynthetic commitment than mRNA production. The mRNA reads and ribosome footprint reads were plotted for each CDS (expressed as edgeR-normalized reads/kb, RPK) for each biological sample (color-coded in Figure 2A). Several features are immediately apparent. While the two parameters are positively correlated (with R^2^ ~ 0.7), the relationship is not strictly linear. Indeed, mRNAs expressed to similar levels can show a large variation in their association with ribosomes. For example, CDSs with mRNA read counts from 500-700 RPK in PCF sample 2 had corresponding ribosome footprint reads ranging from <10 to >6000 (see Figure 2B). Thus, the translational efficiency (TE, calculated as the ratio of ribosome footprint read counts to mRNA read counts for each CDS) of mRNAs varies dramatically even within this narrow range of transcript abundance. In order to compare the sensitivity of ribosome profiling with standard polysome analysis, we examined four single-copy genes with varying TE, but similar mRNA length and abundance in PCF. Northern analysis revealed the distribution of these mRNAs between differently sized polysomes separated by sucrose gradient fractionation of PCF lysates (Additional file 2: Figure S6). Ribosome profiling indicates that Tb927.9.8740 (which encodes the RNA binding protein DRBD3) has a TE of 3.74 in PCF, placing it at the 99^th^ percentile for that stage (see Additional file 2: Figure S2C); while Tb927.1.4690 (which encodes the protein arginine methyl transferase PRMT1) has a TE of 1.10. Both mRNAs were associated with higher-order polysomes, although the four-fold difference in TE is barely detectable on the gradient fractionation due to compression of larger polysomes at the bottom of the gradient. In contrast, Tb927.9.4360 (which encodes the kinetoplast RNA editing ligase KREL1), has a much lower TE (0.29) and peaked in the monosome fraction, although a small amount of association with larger polysomes was seen. Similar results (not shown) were seen for Tb927.8.2650 (which encodes a putative metallo-β-lactamase-like protein), with a TE of 0.09. Thus, as well as providing TE data for essentially all genes, ribosome profiling is more quantitative and has a greater dynamic range than traditional polysome analysis.

It is apparent from Figure 2A that there are two clusters of genes with relationships between translation and mRNA abundance that are distinct from the majority. One group (indicated by the black box) likely represents genes that are expressed at only very low levels, if at all, since both their ribosome footprint and mRNA RPK were <50. As expected, a large proportion of pseudogenes and variant surface glycoprotein (VSG) genes, which are expressed clonally during antigenic variation in BF and not expressed in PCF, fall in this sector (Figure 2C). While many genes encoding *T. brucei*-specific hypothetical proteins also have low ribosome footprint read counts, the majority show higher levels of expression.

The other cluster (indicated by the red circles in Figure 2A) is composed of mRNAs with comparatively low TEs, despite having high mRNA expression levels. We observed that this cluster was comprised almost exclusively of genes corresponding to structural components of the cytoplasmic ribosome (Figure 2D, green dots). While this cluster was always separate from the main set of genes, the displacement varied between samples, both within and between stages. A few other proteins that are not known to be structural components of the ribosome lie within the cluster shown in Figure 2D. These include three genes (Tb927.9.8100, Tb927.9.8130, and Tb927.11.9700) encoding subunits of the nascent polypeptide associated complex, which associates with the ribosome and assists in protein folding [45], and one isoform of eukaryotic initiation factor 5a (Tb927.11.740). Also present are two newly identified genes described as ubiquitin fusion proteins (NTCDSTB.11.NT.154 and 155) [10]. These ubiquitin domains are fused to an RPL40 domain to generate RPL40, a protein of unknown function in the 60S subunit. However, a similar ubiquitin fusion RPS31 is required for the functional integrity of eukaryotic 40S subunits [46]. Another three genes (NTCDS.TB.8.NT.93, 94, and 95) encode identical 34 amino acid proteins that show no conserved domains or sequence similarity, except to closely related species *Trypanosoma vivax* and *Trypanosoma congolense*. We speculate that the functions of these novel proteins may be related to the cytoplasmic ribosome.

### Changes in translation between stages

High level unsupervised clustering of gene level ribosome footprint read counts from all nine samples (three sets of biological replicates of strain 927 PCF and *in vivo*-derived slBF, plus strain 427 cBF) was performed using edgeR. The resulting multidimensional scaling plot showed they fell into three distinct groups, with all BF samples separated from PCF samples by the primary component, and the slBF and cBF samples separated by the secondary component (see Additional file 2: Figure S7). Thus, stage-specific expression changes dominated any strain-specific differences between the two sets of BF samples.

**Figure 3:**
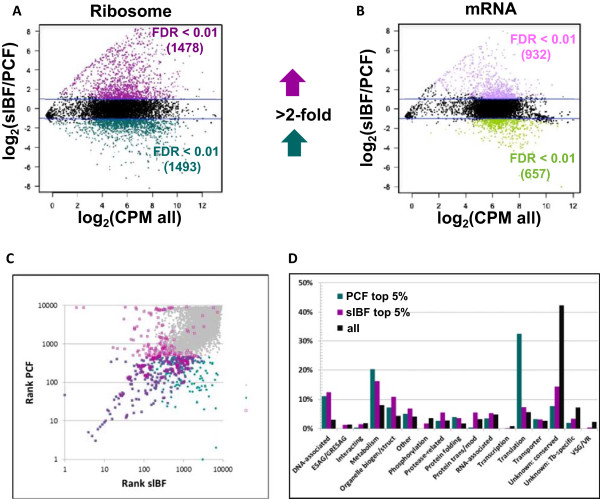
**Ribosome profiling reveals extensive differential protein production.** In this smear plot the fold change in read counts for ribosome footprint **(A)** and mRNA **(B)** were plotted against average read counts per million reads of the pooled libraries for slBF and PCF. Dots that lie outside the blue lines are up-regulated at least 2-fold. Those that are statistically supported (FDR ≤0.01) are colored (green/dark green for PCF and pink/magenta for slBF). Note that almost twice as many genes (2971) up-regulated for protein production as compared to mRNA expression (1589). **C)** Stage-regulation of genes most highly expressed at the level of protein production. This dot plot depicts the gene rank for protein production in slBF and PF. The rank is based on median ribosome footprint RPK in the biological replicates. Those in the top 5% for slBF are outlined in magenta, those in the top 5% for PCF are green, and those that are in the top 5% for both appear purple. The remaining genes are marked in gray. **D)** Categorization of most highly expressed genes compared to genome-wide representation. Top 5% of slBF, magenta; top 5% PCF, green; genome, black.

Gene-level ribosome footprint read counts were compared across biological conditions to assess changes in the translational landscape across stages (Additional file 4 provides comparison data for all genes). As shown in Figure 3A, after excluding the 743 annotated pseudogenes, 1478 of the remaining 8398 genes had at least 2-fold more ribosome footprint reads in slBF than in PCF and 1493 had at least 2-fold more in PCF than in slBF (using a false discovery rate (FDR) <0.01). In contrast, only 932 and 657 showed a statistically significant >2-fold increase in mRNA read counts for slBF and PCF respectively (Figure 3B). Thus, ~35% of all genes showed statistically supported stage-regulated expression of protein production between these two conditions, while only ~19% showed similar changes in mRNA abundance. Of the former, 81% showed similar differences (*i.e.* >1.5-fold change in the same direction) in the cBF to PCF comparison, providing a high level of confidence that changes reflect stage-specific changes. As will be discussed later, the vast majority of genes that were up-regulated in slBF as compared to cBF encoded VSGs or expression site-associated genes (ESAGs, also often associated with antigenic variation in BF), while those that were down-regulated in slBF alone fell into several different functional categories.

Of the mostly highly translated genes (those ranked in the top 5^th^ percentile for ribosome footprint RPK in each stage, 701 in total) in slBF or PCF, 30% are shared between both stages (as indicated by purple dots in Figure 3C). These include α- and β- tubulins, translation elongation factor 1α, aldolase, and glycerol 3-phosphate dehydrogenase. Of the remaining genes (indicated by magenta for slBF and green for PCF), 19% show more than a 10-fold difference in expression levels between the two stages. As expected, these include VSGs and procyclin (the major surface proteins of BF and PCF, respectively). Additionally, 23 of the 56 genes in the top 5% for protein production in slBF, but in the bottom 50% in PCF, encode proteins of unknown function. Similarly, 3 of the 9 genes that are in the top 5% in PCF, but the bottom 50% in slBF, encode hypothetical proteins of unknown function. Thus, these data highlight a set of unstudied genes that may play roles in parasite development. When the most highly expressed genes were separated into functional categories (see Methods), several categories showed differential protein production between slBF and PCF (Figure 3D). Proteins involved in translation were over-represented in among those up-regulated in PCF (including the ribosomal proteins seen in Figure 2D), while those involved in protein transport/modification and degradation were over-represented in those up-regulated in slBF. Proteins associated with DNA (mostly histones), metabolism (see below), organelle biogenesis (tubulins and flagellar proteins), and protein folding (HSPs and T-complex) were over-represented in the genes highly expressed in both stages as compared to their representation in the genome.

**Figure 4:**
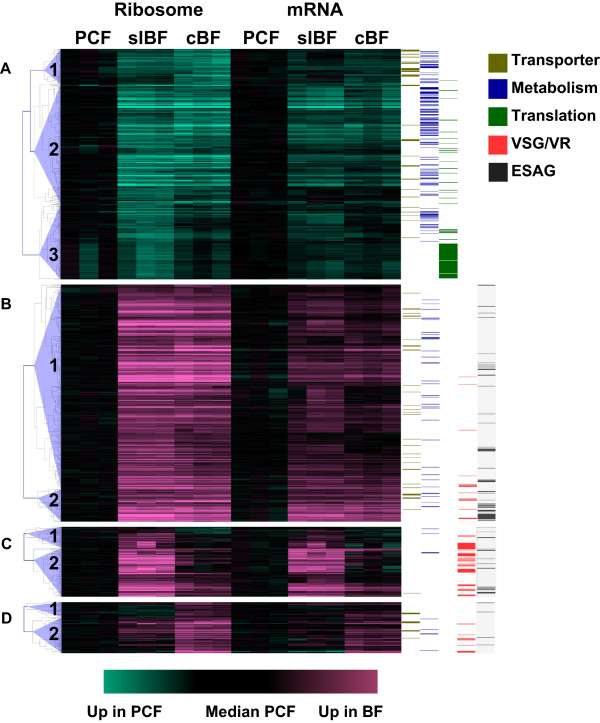
**Cluster analysis reveals distinct patterns of gene expression.** All 1557 genes showing > four-fold change in ribosome footprint edgeR-normalized read counts (with FDR < 0.01 and excluding pseudogenes) between PCF and slBF or cBF were analyzed using MeV (see Methods). The ribosome footprint and mRNA read counts in each of the nine samples were converted to log_2_ fold-change values compared to the corresponding median of the three PCF samples and segregated into four clusters **(A-D)** by K-means (KMC Support), each of which was then separated into 2 or 3 sub-sets by hierarchical clustering. Genes up-regulated in PCF are shown in aqua, while those up-regulated in slBF or cBF are shown in pink. The position of genes encoding transporters (olive), metabolic enzymes (blue), translation machinery (blue), VSGs/VRs (red) or ESAGs (black) are indicated by the colored bars to the right.

To visualize all substantial changes in mRNA abundance and translation between stages, we performed clustering analysis (based on fold-change in mRNA and ribosome footprint read counts) for all genes with at least a four-fold change in ribosome footprint reads between any two of the biological conditions. As shown in Figure 4, these genes segregated into four distinct clusters, each containing 2-3 sub-clusters. Cluster A contains 608 genes that are up-regulated in PCF, while clusters B, C and D contain 627, 185, and 135 genes, respectively, that are up-regulated both slBF and cBF (B), slBF alone (C) or cBF alone (D). Cluster A shows an over-representation of genes involved in metabolism and transport (see Figures 4 and Additional file 2: Figure S10), reflecting the up-regulation of oxidative phosphorylation and amino acid metabolism of PCF compared to BF [47, 48]. In addition, sub-cluster A3 is enriched for the structural components of the cytoplasmic ribosome mentioned above, highlighting the variation in translational activity between PCF and slBF, with intermediate levels in cBF. As expected, clusters B, C and D contain a large number of ESAGs that were up-regulated in both BF conditions (Figures 4 and Additional file 2: Figure S8). Numerous VSG genes are present in clusters C and D, reflecting both antigenic variation and extensive polymorphisms between the strains used. Interestingly, a disproportionate number of transporters, interacting proteins and proteases are up-regulated in either slBF, cBF or both (see Additional file 2: Figure S8). In addition, many genes involved in glycolysis, glycerol and lipid metabolism were up-regulated in BF (see Figure 4), although these categories are not significantly over-represented in clusters B, C or D relative to the entire genome.

### Changes in translational efficiency

**Figure 5:**
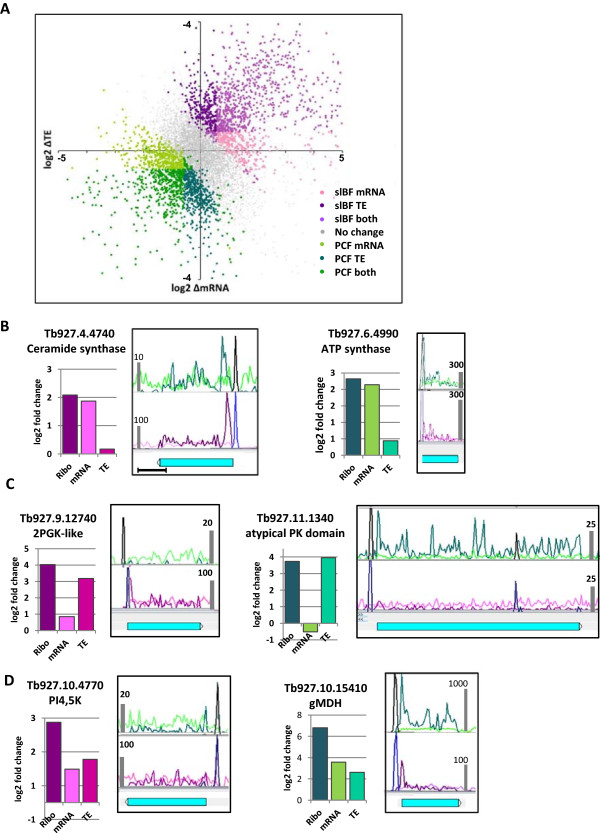
**Regulation of gene expression at the level of TE and mRNA abundance. A)** Genome-wide plot of the change in TE versus the change in mRNA between slBF and PCF, expressed as log2 ratios. **B-D)** Examples of genes where changes in protein production are mediated by different mechanisms. Panel **B**, regulation primarily by changes in mRNA abundance; Panel **C**, regulation primarily by changes in TE, and Panel **D**, regulation in which changes in both mRNA and TE contribute strongly. The histograms show the median log2-normalized fold change in read counts for ribosome footprint, mRNA and the TE. In the histograms, magenta tones are used for genes up-regulated in slBF while green tones are used for those up-regulated in PCF. The Artemis view from PCF3 (green) and slBF3 (magenta) are shown for each gene, with ribosome footprint being the dark color and mRNA being the light color. Similar changes were seen for cBF versus PCF. The genes depicted in this figure had negligible multi-mapping reads. The bars at the edge of the graphs indicate the relative scaling of ribosome profiling and mRNA read counts in the two stages. The SL reads (black in PCF, blue in slBF) are not to scale. The scale bar below the first panel represents 500 nt, used for all images).

Increased protein synthesis can be mediated by a change in mRNA level or translation efficiency, or a combination of both. Changes in mRNA levels are well known to be important during *T. brucei* development, with studies using different technologies and statistical cutoffs yielding estimates of 5-6% ([6, 9], ~25% [7] and ~40% [11] of genes as being differentially expressed between cBF and PCF. However, changes in protein production (as measured by ribosome profiling) are generally higher than those in mRNA abundance, providing evidence for changes in TE being involved in regulation of differential gene expression. This is the case in terms of both the number of genes that were significantly differentially expressed between PCF and slBF (2971 ribosome footprint *vs* 1589 mRNA, see Figure 3A and B) and the magnitude of the change for individual genes (see Figure 4). Indeed, the sub-clusters in Figure 4 begin to segregate genes with similar changes in translation (ribosome footprint) and mRNA (sub-clusters A2, B2, C2, and D2), from those where the change in translation was much greater than that in the mRNA level (sub-clusters A1, A3, B1, C1 and D1), although the separation is not complete. This variation in the contribution of changes in mRNA abundance and changes in TE to yield differences in protein production can be more readily seen by plotting these parameters for all genes (Figure 5A). In this representation, the grey dots (around the downward diagonal) represent genes for which there was no significant change in protein production (even though in some cases the mRNA level or TE may change), while the colored dots correspond to genes with at least a 2-fold change. The light-colored dots near the x-axis indicate genes where changes in mRNA levels accounted for most (or all) of the change in translation, while the dark-colored dots near the y-axis represent genes where most of the change in translation was mediated by TE. There are also a large number of genes (indicated by the medium-colored dots near the upward diagonal) where both mechanisms appeared to play an important role.

We have identified a number of clear examples for each of these three categories of regulation. Two cases that illustrate regulation (primarily) by mRNA are shown in Figure 5B. The mRNA from Tb927.4.4740, which encodes a ceramide synthase-related protein, increased 3.6-fold in slBF (compared to PCF), accounting for most of the 4-fold increase in protein production. Similarly, the mRNA from Tb927.4990, which encodes the δ-subunit of ATP synthase, was 4.4-fold higher in PCF, with a corresponding 5-fold increase in ribosome footprint read counts. Since most of the increase in translation was due to the change in mRNA abundance, the extent of protein production specified by these genes can be readily assessed by RNA-seq alone. However, for the other genes, RNA-seq provides only a partial (or even misleading) picture. Figure 5C shows two examples of regulation mediated primarily by change in TE. Tb927.9.12740 (which encodes a protein with similarity to 2-phosphoglycerate kinase) had ~16-fold higher ribosome footprint read counts in slBF, while the mRNA read count increased by only ~1.7-fold. Similarly, the mRNA read count from Tb927.11.1340 (which encodes a protein with an atypical protein kinase domain) was slightly (1.3-fold) lower in PCF, but the ribosome footprint read count increased by ~13-fold. Thus, in both cases the change in TE (calculated to be ~9-fold and ~16-fold, respectively) accounted for most (or all) of the increased translation. Finally, the two cases in Figure 5D provide examples of where a combination of changes in both mRNA abundance and TE appear to play a role in regulating gene expression. Tb927.10.4770 (which encodes phosphatidyl inositol 4,5 kinase) showed a 5-fold increase in ribosome footprint reads in slBF, resulting from a 2-fold increase in mRNA reads and 2.4-fold increase in TE; while Tb927.10.15410 (glycosomal malate dehydrogenase) had 111-fold more ribosome footprint reads in PCF, resulting from a ~12-fold increase in mRNA and ~6-fold increase in TE.

In order to systematically and statistically assess the role of mRNA and TE changes in regulating gene expression, we applied a generalized linear model (GLM) framework within DESeq [49] to the raw CDS read count data from both mRNA and ribosome footprint. The GLMs corresponded to the potential regulatory mechanisms based on: a) mRNA abundance change only; b) TE change only; c) both mRNA abundance and TE change and d) no significant regulation. DESeq tested each gene individually for the fit to the models, and those that showed significant regulation were then assigned to model a, b, or c, as described in Methods. The GLM results, alone and in combination with edgeR criteria (2-fold difference in ribosome footprint reads, FDR < 0.01), are shown in Table 1. The results of these analyses for slBF *versus* PCF indicate that TE (either alone or together with changes in mRNA level) plays a substantial role in regulating stage-specific gene expression, accounting for 805/1290 (62%) of the genes that were more highly expressed in PCF and 996/1359 (73%) of those more highly expressed in slBF. However, only a modest number of genes (59 in PCF and 126 in slBF) are predicted to be regulated by TE alone, although these analyses are likely complicated by the complex relationship of translation to mRNA decay [50], which may over-emphasize the role of changes in mRNA abundance. Importantly, when the same analyses were applied to cBF *versus* PCF, we obtained similar results (Table 1), except that (as indicated above) there were fewer stage-specific changes in gene expression in this situation, especially in terms of genes that were expressed at higher levels in PCF. Nevertheless, 769 genes were identified where GLM analysis showed that regulation of TE plays a significant role in stage-specific gene expression for both slBF and cBF as compared to PCF and for which edgeR analysis also showed significant stage-specific changes in ribosome footprint (≥2 fold, FDR < 0.01). These included at least 33 cases where there was no significant change in mRNA level (Table 2).

**Table 1:** **Mechanisms of gene regulation**

slBF:PCF	Stage^a^	DESeq	Both DESeq and edgeR^b^	%
no change	-	5212	5749	68.5%
TE only	PCF	62	59	0.7%
both	PCF	872	746	8.9%
mRNA only	PCF	654	485	5.8%
TE only	slBF	130	126	1.5%
both	slBF	1023	870	10.3%
mRNA only	slBF	445	363	4.3%
**cBF:PCF**	**Stage**	**DESeq**	**Both DESeq and edgeR**	**%**
no change	-	6528	6671	79.5%
TE only	PCF	16	15	0.2%
both	PCF	607	538	6.4%
mRNA only	PCF	212	185	2.2%
TE only	cBF	124	124	1.5%
both	cBF	737	714	8.5%
mRNA only	cBF	174	151	1.8%

^a^stage with higher protein production.

^b^concordance of edgeR analysis indicating 2-fold up-regulation for ribosome footprint read counts (FDR <0.01) and DESeq GLM model. Those that were not concordant were binned into the “no change” group in this column.

**Table 2:** **Genes with stage-regulated expression controlled primarily by TE** ^**a**^

GeneID	Product	Log2TE slBF-PCF^b^	Log2TE cBF-PCF
Tb927.1.1580	cytochrome c oxidase assembly factor SCO1/2	−2.92	−2.10
Tb927.1.1820	PIN nuclease domain protein	2.43	2.12
Tb927.1.3130	protein kinase	2.38	3.46
NTCDS.Tb3.NT.15	hypothetical protein	2.62	2.61
Tb927.4.2290	glucose transporter	1.95	2.22
Tb927.4.5190	hypothetical protein, conserved	−3.11	−3.54
Tb927.5.285b	receptor-type adenylate cyclase ESAG4	−2.24	−2.26
Tb927.5.320	receptor-type adenylate cyclase GRESAG4	−2.00	−2.16
Tb927.5.430	ISG65/75 domain protein	2.47	1.69
Tb927.7.1840	zinc finger protein	2.70	1.97
Tb927.7.1880	zinc finger protein	2.82	1.99
Tb927.7.6150	hypothetical protein, conserved	3.65	3.52
Tb927.8.2480	3-oxo-5-alpha-steroid 4-dehydrogenase-like	2.97	2.69
Tb927.8.2780	RNA-binding protein RBP10	4.75	7.35
Tb927.8.4570	RING domain protein	2.39	1.88
Tb927.8.5480	hypothetical protein, conserved	2.10	2.19
Tb927.8.6130	hypothetical protein, conserved	2.63	1.95
Tb927.8.7500	hypothetical protein, conserved	2.18	2.45
Tb927.9.1500	protein kinase	1.58	3.52
Tb927.9.3100	hypothetical protein, conserved	2.09	2.57
Tb927.9.3820	syntaxin	1.88	2.03
Tb927.9.15850	hypothetical protein	3.67	3.30
NTCDS.Tb9.NT.51	hypothetical protein	2.53	2.49
Tb927.10.2210	ubiquitin carboxyl-terminal hydrolase	−2.90	−3.07
Tb927.10.12500	P-type H -ATPase	−2.08	−2.29
Tb927.10.14910	sarcoplasmic reticulum sarcalumenin	1.63	2.11
Tb927.11.1340	Protein kinase-like domain protein	−3.96	−2.71
Tb927.11.3630	nucleobase/nucleoside transporter 8.1	4.28	2.16
Tb927.11.7820	endonuclease/exonuclease/phosphatase	1.94	3.12
Tb927.11.12730	hypothetical protein	3.00	2.73
Tb927.11.14740	Recombinase-like domain protein	1.70	2.08
Tb927.11.15840	L-Lysine transport protein	1.09	2.11
Tb927.11.15860	L-Lysine transport protein	1.14	2.05

^a^GLM and edgeR both indicate TE plays a significant role and that mRNA change is not significant.

^b^Positive values indicate higher TE in BF, negative indicate higher TE in PCF.

To determine whether different classes of genes were regulated by TE *versus* mRNA during parasite development, we grouped them into the broad functional categories described above (see Additional file 1: Table S4 and Additional file 2: Figure S9). As mentioned above, cytoplasmic ribosomal proteins (a subset of the “Translation” category) were over-represented in those genes more highly expressed in PCF, and TE appeared to play a prominent role in their regulation, although some genes in the Translation category were also regulated by mRNA abundance. In contrast, genes encoding metabolic enzymes and proteins involved in protein folding that were up-regulated in PCF appeared to primarily use mRNA abundance to regulate their expression. Among the genes up-regulated in slBF, mRNA level appeared more important for VSG and ESAGs, while transporters were regulated more by change in TE. However, almost half of the genes that were regulated by TE alone encoded proteins with unknown (but conserved) function. We also observed that genes with the highest levels of protein production were more likely to exhibit stage-regulation (Additional file 2: Figure S10).

### uORFs and translational regulation

In other eukaryotes, one of the mechanisms by which gene-specific changes in translation can be exerted is through the presence of uORFs that interfere with the translation of the main CDS [27, 51–53]. Not all uORFs modulate translation, but evidence indicates that those that do act through being themselves translated (only some uORFs are efficiently translated) [27, 51–53]. Initiation at a uORF can compete with initiation at downstream translation start sites, reducing translation of the main CDS. Alternatively, increased translation of a uORF that overlaps with the main CDS could also interfere directly with the initiation of other ribosomes at the main translation start site.

**Figure 6:**
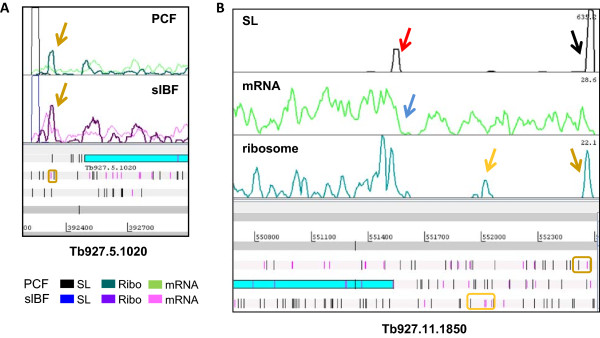
**Predicted uORFs and translation. A)** Example of a translated uORF that convincingly lies on the same transcript as the main CDS. A 5 aa uORF outlined in gold and delineated by the start codon (pink line) and a subsequent stop codon (black line) in the reading frame 2 is associated with the main CDS of Tb927.5.1020. It is translated in both stages (RRS = 23.5). We do not see a significant difference in TE of the main CDS between stages (slBF:PCF Δlog2 TE = -0.25). **B)** Candidate uORFs may not not lie on the same transcript as the CDS of Tb927.11.1850. Predicted uORFs are seen in all three reading frames downstream of the computationally predicted 5′ end of the mRNA defined by a peak in SL reads as described in Methods (black arrow). Two of these uORFs bear ribosome footprints with RRS scores >70 (gold arrows; ORFs are outlined on the map). The blue arrow marks a dip in the mRNA levels, followed by a second trans-splicing site just before the main CDS (red arrow). Thus, it is not convincing that most transcripts bearing the Tb927.11.1850 CDS also bear these putative uORFs. Data are shown for PCF3, although similar profiles were seen with slBF.

Analysis of the 5′ UTRs for all genes using our 5′ end mapping indicated that only 950 intact genes had potential uORFs, for a total of 3284 uORFs (see Additional file 5 for coordinates and read counts, and Figure 6A for example). The percentage of genes is smaller than that cited in a previous study [20] (11% vs 22%) , likely due to the further refinement of 5′ UTRs that we performed (see Methods). Additionally, manual inspection of a subset indicated that some putative uORFs we identified cannot be confidently placed on the same transcript as the CDS, since there was an intervening SL site and the mRNA reads dropped to near-baseline just prior to this site (see Figure 6B for example), suggesting that this likely represents an over-estimate of the number of genes with genuine uORFs. A similar phenomenon has been observed in budding yeast when 5′ terminus sequencing and ribosome profiling data were combined to discover apparent uORFs that actually reflected distinct short transcripts [54].

Of the 2646 uORFs that did not overlap or abut the main CDS, RRSs could be calculated for only 458; the remainder have very low ribosome read counts on the uORF or read counts of zero for other values (see Methods and Additional file 5). A total of 322 genes had uORFs with an RRS > 2, providing some indication of uORF translation (one example is shown in Figure 6A). Furthermore, as compared to mRNAs with no uORFs, mRNAs that contained putative uORFs with RRSs >2 showed ~2-fold lower TEs and mRNA read counts (with p < 10^−48^) in all three biological conditions (see Table 3) and in mRNA RPK (not shown). The TE difference between the two groups persisted even when we accounted for difference in mRNA read count levels by analyzing only genes with mRNA read counts in the second and third quartiles (Table 3). Although we could not calculate the RRSs of uORFs that overlap the main CDS, these CDSs also showed a similarly low TE (not shown). Thus, it is likely that uORFs reduce translation of a number of mRNAs in trypanosomatids, as in other organisms. Analysis of the functional category of uORF-containing mRNAs revealed nothing remarkable, except for ~2-fold over-representation of *T. brucei*-specific genes and slight (<2-fold) under-representation of those involved in translation, transport, organelle biogenesis/structure, and proteolysis, as well as VSGs.

**Table 3:** **The presence of uORFs and TE**

	PCF		slBF		cBF	
All^a^	*uORF* ^*b*^	*no uORF*	*uORF*	*no uORF*	*uORF*	*no uORF*
Observations	322	7009	322	7009	322	7009
**TE**						
Mean	0.3756	0.8434	0.3092	0.8031	0.3647	0.8678
P^c^	1.9e-49		7.5e-65		2.7e-54	
**mRNA**						
Mean	522.4	1013.5	599.4	878.3	555.8	891
P	1.9e-07		1.2e-07		7.6e-06	
**mRNA (q2 + q3)** ^**d**^	*uORF*	*no uORF*	*uORF*	*no uORF*	*uORF*	*no uORF*
Observations	186	3476	161	3529	181	3512
**TE**						
Mean	0.3544	0.7239	0.3007	0.7648	0.3308	0.6912
P	1.7e-28		7.9e-36		1.4e-29	
**mRNA**						
Mean	552.8	558.2	568.4	571.9	584.7	570.5
P	0.6514		0.8048		0.1144	

^a^Excluding pseudogenes and genes with unknown 5′ UTRs.

^b^Those genes with uORF RRSs > 2.

^c^Mann Whitney U calculation.

^d^The genes were further restricted to those with mRNA levels in quartiles 2 and 3 for the biological condition analyzed (PCF 323-857, slBF 334-902; cBF 350-851 median mRNA read counts).

We saw no enrichment for genes with uORFs among those that were classified by GLM as stage-regulated by TE (alone or in concert with regulation of mRNA abundance) (Additional file 2: Figure S11). Of the 13 genes that were regulated primarily by TE and had putative uORFs, five showed somewhat higher TE in the stage in which the uORF had more ribosome footprint reads, while five had very few uORF reads in both stages, two lacked a true uORF, and one was extensively multi-mapping precluding further analysis. Thus, we were unable to find convincing evidence that the putative uORFs contribute to stage-regulation of translation between PCF and slBF. This data does not rule out the possibility that some uORFs confer a component of stage-regulation, which could be revealed with further refinement of the transcriptome and translatome or by examination of other developmental stages.

## Conclusions

The sequencing of the *T. brucei* genome in 2005 [55] ushered in an era of high-throughput analyses of the transcriptome [5–7, 9–11] and proteome [2, 3] of both insect and mammalian stages of this parasite. While these studies have been very informative and are transforming trypanosomatid research, both approaches have shortcomings that limit their usefulness for researchers in the field. Microarray and subsequent RNA-seq analyses have elucidated numerous changes in mRNA levels between life cycle stages at a comprehensive genome-wide scale, but they cannot identify genes that are regulated at the level of translational control. Conversely, mass spectrometry-based proteomic analyses suffer from lack of coverage, interrogating less than half of all cellular proteins. The recently developed technique of ribosome profiling [36, 37] covers the middle ground by quantitatively interrogating mRNAs for the presence of ribosomes, thereby revealing the rate of translation for every gene. This ribosome-centric approach provides more specific quantitation and greater dynamic range than the mRNA-centric technique polysome profiling (coupled with microarray or other genome-wide analysis), although it does not reveal distinct pools of mRNA that can be observed using latter approach.

Comparison of our results with those obtained from the most comprehensive published proteomic analysis comparing BF and PCF [2, 3, 56], shows a good correspondence between changes in translation and protein level (see Additional file 2: Figure S12). Most (84%) of the proteins that showed >2-fold up-regulation in BF had at least 1.5-fold up-regulation in protein production in one or both BF conditions used in this study. There was slightly less agreement (63%) for proteins up-regulated in PCF, perhaps reflecting differences between strains and growth conditions used in the two studies. Some discrepancies are to be expected since the proteome is also modulated by individual protein stabilities, which would not be reflected in our data. While it is known that most abundant proteins are quite stable in PCF trypanosomes [57], it is also likely that a subset of proteins are less stable, such as those required at specific points in the cell cycle. It is also interesting to note that changes in protein production had a greater magnitude than those in protein abundance, perhaps reflecting a larger dynamic range and sensitivity for ribosome profiling. This enabled us to detect 529 genes with a 10-fold or greater change in protein production between stages, of which only 143 were detected by the proteomics approach.

While this manuscript was in preparation, another paper describing the application of ribosome profiling to *T. brucei* was published [20], including a comparison of single samples of cBF and PCF. That study revealed extensive changes in translation during parasite development, but lacked the biological replicates to provide a robust statistical analysis of the stage-regulated changes. Here, we compared three biological replicates of PCF from one strain (927) to three replicates of both *in vivo* derived slBF from the same strain and cBF of another strain (427). When contrasting the two studies, R^2^ values of ~0.63 were seen when comparing PCF and cBF ribosome footprint data, with stronger correlations for mRNA data (R^2^ of 0.68 and 0.85 for PCF and cBF respectively) (Additional file 2: Figure S13A). Of the 27 genes specifically noted by Vasquez et al. [20] as showing the highest level of translational regulation, 24 showed at least a 2-fold change in TE in our comparison of slBF vs PCF. Conversely, over 65% of genes with at least a 4-fold increase in TE in BF in our experiments also showed at least a 2-fold change in the published study (Additional file 2: Figure S13B). Given the likely differences in growth conditions, strains, and data analysis these similarities strengthen the conclusions that translational regulation is important in the development of these parasites. Moreover, our use of biological replicates revealed several phenomena that were not previously apparent.

Firstly, we observed that while the mRNAs for genes encoding structural components of the cytoplasmic ribosome were relatively abundant under all conditions, their translation efficiency varied considerably between samples, both within and between conditions, and they were relatively poorly translated. Low rates of translation for mRNAs encoding ribosome-associated proteins is a well-known phenomenon in other organisms, including under conditions of cell stress [58, 59]. This may explain the somewhat surprising observation that the TE of ribosomal proteins was lower in slBF than PCF, since although slBF grow more rapidly than the cultured insect form, they are exposed to stresses *in vivo*. Additionally, the commitment of some slBF parasites to exit the cell cycle to become stumpy forms (even though the populations were >95% morphologically slender) may contribute to the reduced translation of ribosome proteins. This argument is buttressed by the finding of an intermediate TE for ribosomal proteins in cBF derived from a monomorphic strain (*T. brucei* 427), which does not differentiate into stumpy forms *in vitro* or *in vivo*, but are nonetheless highly sensitive to cell density. Thus reduced translation of ribosomal proteins in *T. brucei* may be an early event when proliferation slows. In many mammalian cells and in maize, regulation of ribosomal protein production appears to rely on pyrimidine rich elements at the 5′ end of the mRNAs known as TOP elements [59–61]. However, in *T. brucei* polypyrimidine tracts are signals for *trans*-splicing (and are removed during processing) and mRNAs are all identical at their 5′ termini. We examined the 5′ UTRs of the cytoplasmic ribosomal proteins and saw no enrichment of pyrimidines (43% CT as compared 45% for all transcripts, exclusive of the common 5′ SL sequence, see Additional file 2: Figure S14). Thus, the mechanism of translational regulation of these structural proteins of the ribosome must differ in trypanosomes. However, we detected no enriched motifs in the 5′ UTRs as compared to the overall transcriptome (by MEME analysis, [62]), although the ribosomal protein 5′ UTRs are shorter than average (median of 21 nt as compared to 87 nt median for all genes). As regulation of ribosome biogenesis is a central part of stationary phase development in many organisms and if the trypanosome must modulate the TE of ribosomal proteins during its life cycle, then the mechanisms underlying this regulation, which appear to differ from the analogous control of animal ribosomal protein translation, may present a therapeutic target. Future studies dissecting the mechanism of this control in trypanosomes will therefore prove interesting.

Our results also revealed that regulation of protein production in *T. brucei* is more extensive than previously anticipated from changes in mRNA abundance. While just under two hundred genes appeared to be regulated by changes in TE alone, several thousand show changes in translation substantially larger than the changes in their mRNA level. It is possible that some of these changes in TE reflect alternative splicing, which can be further investigated using existing and our updated SL data, although additional experimentation will be required. It will also be interesting to compare ribosome profiling of slBF with stumpy BF, which are growth-arrested forms that are poised for transformation into PCF upon ingestion by the tsetse fly. Previous microarray analyses [6, 7], demonstrated that few mRNAs differ in abundance between stumpy BF and slBF, but early studies indicated that translation is much reduced in stumpy BF [63] and microarray analysis of polysome fractions has identified a subset mRNAs that are differentially translated between the two stages [19]. Ribosome profiling has greater sensitivity in revealing changes in protein production than does polysome analysis, so we might expect that under conditions of limited translation (such as stumpy BF), more genes will be revealed as translationally regulated. By analysis of different additional stages and conditions, it is likely that different groups of genes under translational control will be revealed, potentially operating through different mechanisms.

## Methods

### Parasites and cell extracts

The pleiomorphic *T. brucei* strain TREU927, which has the most complete genome sequence at present [55], was employed for production of slBF and PCF. Three biological replicates of each stage were used (see Additional file 1: Table S1). slBF were grown in irradiated Wistar rats following injection of 10^8^ parasites derived from stabilates following IACUC approved protocols. The parasites were harvested on day 3 at a parasitemia of 5 × 10^7^-1 × 10^8^ cells per ml. Only parasite populations with greater than 99% slender cells were used. After harvest, the blood was centrifuged and the buffy coat extracted and placed into 20 ml HMI-9 medium (without serum) pre-warmed to 37°C. To arrest translation, cycloheximide was added to 100 μg/ml and incubated for 2 minutes at 37°. To rapidly chill the cells, 300 ml of ice-cold phosphate buffered saline with glucose (PSG) was added and the cells were pelleted at 4°. Parasites from 2-3 animals infected from the same culture of *in vitro* grown parasites were pooled and lysates prepared as described below. Microscopic analysis showed that rat white blood cells represented less than 1% of the population. For cBF, a derivative of *T. brucei* monomorphic strain Lister 427 was grown *in vitro* in HMI-9 medium [64] and harvested when the cultures were between 8 × 10^5^ and 1.6 × 10^6^ parasites/ml. The cultures were centrifuged for 10 minutes at 900 × g, resuspended in 25 ml of pre-warmed serum-free medium, treated with cycloheximide and rapidly chilled as above. Three biological replicates of these *in vitro* derived cBF were used for comparative purposes. We grew strain 927 PCF in SDM79 medium containing glucose [65], with 2-4 × 10^9^ parasites being harvested in mid-log phase (density of 5 × 10^6^-1.2 × 10^7^ cells/ml). The initial large volume of culture was centrifuged at 5000 × g for 5 minutes at room temperature and the pellet resuspended in 50 ml of medium lacking serum. The parasites were incubated for 2 min in cycloheximide as above, rapidly chilled by the addition of 250 ml PSG, and collected by centrifugation.

Cell pellets were resuspended in Buffer A (10 mM Tris pH 7.4, 300 mM KCl, 10 mM MgCl_2_, plus protease inhibitors [63]) to approximately 1.3 × 10^9^ cells/ml. Approximately one-third of the sample was placed into TRIzol (Life Technologies) for RNA extraction following the manufacturer’s suggested protocol. To the remainder, one-sixth volume of buffer A containing 0.2M sucrose and 1.2% Triton N-101 was added and the samples were homogenized (30 strokes using a chilled dounce with a 0.004-0.006 inch clearance pestle). After transfer to a pre-chilled microfuge tube, the samples were clarified by centrifugation in a microfuge at 15,000 rpm for one minute. The supernatant was withdrawn, pooled if needed, and then aliquots flash frozen in liquid nitrogen for storage at -70°C. These extracts were then used for ribosome footprinting or polysome gradients*.* Polysome analysis was performed as previously described [63].

### Library preparation and sequencing

#### Ribosome footprinting

Preliminary experiments established the appropriate conditions for RNAse I treatment of lysates (Additional file 2: Figure S1A). After thawing on ice, RNase I (Ambion) was added at 30 units/OD_260_ of lysate. Samples were then incubated for 1 hour at room temperature. RNase digestion was stopped by adding 400 units RNasin (Promega). Samples were them layered over a 1 ml 1M sucrose cushion prepared in buffer A and ribosomes were pelleted by centrifugation for 4 hours at 70,000 × g in an SW55 rotor. After removing the supernatant, the ribosomal pellet was resuspended in 500 μl buffer A with 10 mM EDTA replacing the MgCl_2_ to dissociate the ribosomes (Additional file 2: Figure S1B). The protected fragments were then separated from contaminating larger ribosomal RNA fragments by passage through an Amicon Ultra-4 or YM-100 column with 100,000 MW cut-off. The RNA in the flow-through (400 μl) was extracted with phenol:CHCl_3_:isoamyl alcohol and the RNA precipitated.

#### mRNA libraries

Poly(A) + RNA was isolated using Dynabeads mRNA Direct (Life Technologies). RNA was fragmented as described [36] and fragments between 30 and 70 nucleotides isolated. For a detailed protocol on generating sequencing libraries for both the ribosome protected and fragmented mRNA library see Ingolia *et al*. [66]. Briefly, following dephosphorylation the adapter Linker-1 (IDT) was ligated to the 3′ end of the fragment and the ligated product gel purified. The adapter was used for priming reverse transcription with the primer RP_index_RT (all primers are provided in Additional file 1: Table S6). Following gel purification the cDNA was circularized with Circ Ligase (Epicenter Biotechnologies). Circles containing ribosomal RNA were subtracted using biotinylated primers at 10 μM. The final library was generated by PCR using RP_index_PCR_forward and one of the RP_index reverse primers.

#### SL RNA-seq libraries

Libraries enriched for the 5′ ends of mRNAs were constructed from three biological samples of strain 927 (two PCF and one slBF of the biological samples above) and one cBF sample from strain 427, as described previously [67]. In brief, RNA was prepared and cDNA synthesized using primer Random5. Second strand synthesis was primed using SL_2^nd^ primer3, which matches the 3′ *T. brucei* SL sequence. The sequencing library was generated by PCR using the primer Multi-PCR P2 and one of the RP_index_PCR_reverse primers.

All libraries were sequenced using Illumina GA II machines at the High Throughput Genomics Unit at the University of Washington to generate ~36 nt reads using the proprietary Illumina read 1 sequencing primer (Rd1 SP) for fragmented mRNA and ribosome profiling libraries, or a custom sequencing primer (SL_SEQ_Primer2) for the SL RNA-seq libraries, as well as the Illumina indexing sequencing primer (Index SP).

### Bioinformatics

Reads were assessed for their average quality, average GC, base composition, and variability between clusters, and those with average quality less than 30 were removed. *T. brucei* strain 927 genome sequences and gene annotations (version 5.0) were downloaded from TriTrypDB. This version of *T. brucei* genome consists of 11 large chromosomes, plus a variety of other contigs (a number of short BAC contigs, 1 bin chromosome for Chr9, and two fork chromosomes for Chr11). We determined that >95% of the genes present in these other contigs are repetitive genes and sub-telomeric genes that were already present on one of the main chromosomes, so only the 11 large chromosomes were considered for the read alignment, as the other contigs would only increase ambiguity in the alignment without providing any extra information.

The fast sequence files were aligned against the 11 chromosome sequences using Bowtie2 [68] in local mode (which allowed us to avoid trimming of adapter sequences from ends prior to alignment), using the following parameters: -D 20 –R 3 –N 1 –L 20 –I S,1,0.50. This allowed a maximum of one mismatch within a 20 nt seed region and a maximum of two mismatches across the entire alignment. The BAM files were sorted, indexed and reads mapping to structural RNAs were segregated in a separate BAM file to allow convenient viewing of the remaining reads in Artemis [69]. RNA sequencing data for ribosome footprint, mRNA, SL mapping has been deposited in the GEO database with the accession number GSE57336, with details of the biological samples deposited in the Bioproject database under accession number PRJNA246300. The RNA-seq data and revised annotations have been provided to TriTrypDB and Wellcome Trust Sanger Center respectively to be integrated into their genome databases.

The periodicity of ribosome footprinting was assessed as follows. For each codon (or in-frame triplet for those positions upstream of the start codon), the percentage of reads commencing at the first, second, or third position of that codon is plotted. Read counts for each codon/triplet were the sum of all 28 and 29 nt reads for all CDSs in specific libraries. Similar calculations were performed for mRNA reads.

#### Defining the 5′ end of transcripts

Reads from SL RNA-seq libraries from PCF1 and slBF1 biological samples (16.8M reads and 14.4M reads respectively) were aligned against the genome using Bowtie with following parameters (-e 70 -l 23 –n 2 –M 1). The major SL site (*i.e*., that with the most mapped reads upstream of the stop codon) for each CDS was identified, as well as other predominant SL sites located further 5′. The 5′ boundary of each mRNA was defined the most 5′ site with a read abundance of at least 40% of the major SL site, and a minimum of 20% of all SL sites for that CDS (from stop codon of upstream gene to the stop codon of the gene of interest). However, if there was an obvious gap in the mRNA read coverage, upstream SL sites were not considered and the next SL site was used. Of the 8398 intact genes, 96% were assigned 5′ UTRs and 84% of these we considered high confidence (defined of at least 50% of the SL reads for that gene, with at least 20 reads at that site). These included 904 high confidence 5′ UTRs for genes lacking defined 5′ UTRs in TriTrypDB and 758 high confidence changes versus those listed in TriTrypDB. A list of the mapped 5′ UTRs used in this study is provided in Additional file 6.

#### CDS refinement

An iterative process used to refine the CDSs in the *T. brucei* strain 927 genome prior to the final assignments of read counts to individual genes. We manually inspected each chromosome for discrepancies from the annotated genome by visualizing SL, mRNA, and ribosome footprint reads from slBF and PCF libraries. New CDSs were added when ribosome footprint reads mapping throughout an ORF that commenced with a canonical start codon and was accompanied by an upstream SL and by mRNA reads (coordinates are provided in Additional file 3). Of these 120 corresponded to CDSs on recently discovered transcripts [10] and were given provisional GeneIDs commencing with “NTCDS”. And additional 62 novel CDSs were also defined and were given provisional GeneIDs commencing with “NCDS”. Boundaries for 573 CDSs were extended or shortened based on SL mapping of the start of the transcript by using the 5′-most in-frame start codon after the major SL site unless ribosome profiling or mRNA levels indicated otherwise. Many of these revised start codons had been previously noted [9, 11], but had not yet been incorporated into the gene models in TriTrypDB v5.0. A comparison of these datasets is provided in Additional file 7. Briefly, 249 matched both of the other datasets and 237 matched one of the other datasets. A total of 249 CDSs were changed in only one dataset and merit further study. We deleted all CDSs annotated as “hypothetical protein, unlikely”, as well as some annotated as conserved hypothetical proteins, that had no evidence of coding potential, according to one or more of the following criteria: 1) most mRNA reads mapped to the wrong strand, 2) the CDS mapped to the 3′ UTR of another gene, showed similar mRNA read depth and lacked its own SL reads, 3) the CDS contained significant gaps in mRNA coverage, and/or 4) contained abundant internal SL reads (listed in Additional file 8). Genes that lacked sufficient information for discrimination were not eliminated; most of these lay within strand switch regions or close to the ends of chromosomes. These changes will be further discussed in another manuscript.

RRSs were calculated for all CDSs based on raw reads as described [38] and are provided in Additional file 3. The 3′ UTR was defined as in TriTrypDB, or if data were lacking, as 100 nt downstream of the stop codon, or to nt -13 relative to the next CDS, whichever was smaller. Briefly, for each CDS, the summed read counts across all ribosome profiling libraries and across all mRNA libraries were used to calculate as follows: (CDS ribosome footprints/3′ UTR ribosome footprints)/(CDS mRNA/3′ UTR mRNA). Only reads completely contained in the relevant region were included. For cases in which no ribosome footprints were seen in the 3′ UTR, a value of 1 was substituted (to avoid divide-by-zero errors and yield a minimal RRS). For cases in which no mRNA reads were obtained for either the CDS or 3′ UTR, an RRS cannot be computed.

#### Normalization and read count assignment

By analysis of the peak of ribosome footprint reads at the start codon, we determined that the footprint extended 12 nt upstream of the first base of the codon being translated (Additional file 2: Figure S3), as previously observed in *Saccharomyces cerevisiae* [36]. Read counts for each CDS were generated using the HTSeq package [70] and included those from nt +46 (1 being the A of the ATG start codon) to the stop codon. For the 5′ UTR, reads from the 5′ end of the mRNA (excluding the SL) to nt -16 were included. Reads partially overlapping CDS or 5′ UTR regions were included in the counts. The percentage of multi-mapping reads for each CDS is provided in Additional file 3. Raw read counts from each library type (mRNA, ribosome profiling) and each region (CDS region and UTR region) were tested for their differential expression in PCF, slBF and cBF samples using edgeR [40] and all nine biological samples (PCF, slBF, and cBF) were normalized coordinately. To avoid over-emphasizing fold-changes for genes with very low/zero read counts, 5 reads were added to the count for each CDS and 5′ UTR region prior to normalization. edgeR was also used to correct for sample-specific variation, normalizing the data based on sequencing depth (total read counts per sample) and RNA composition (weighted trimmed mean of log expression ratios -TMM). These normalized read counts were used in our further TE calculations and unpaired t-tests were used to estimate variation within biological replicates. As a second means of estimating variation within biological replicates, the negative binomial model was fit under GLM framework onto the raw reads, along with corresponding scaling factors for each sample. From this fit, common, trended and gene-wise dispersions (biological coefficient of variation) were estimated using the corresponding functions in edgeR. The raw read counts along with sample specific scaling factors, gene-wise dispersions were fed into edgeR’s GLM framework and a GLM likelihood ratio test was performed to identify differentially expressed genes.

#### Cluster analysis of changes in gene expression

All CDSs (excluding pseudogenes) that showed > four-fold change in ribosome footprint edgeR-normalized read counts, with statistical support of FDR (Benjamini and Hochberg method) <0.01, in pairwise comparisons between PCF, slBF and/or cBF were analyzed using MeV [71]. The ribosome footprint and mRNA read counts for these 1559 CDSs from each of the nine samples were converted to log_2_ fold-change values compared to the corresponding median of the three PCF samples before being imported into MeV. The genes (but not samples) were segregated into clusters using KMC Support with the following parameters: Distance Metric = Pearson Correlation; Means or Medians = K-means; K-means repetitions = 50 runs, with 80% threshold for occurrence in the same cluster; K-means run parameters = 4 clusters, 50 iterations. Hierarchical Trees were also constructed using HCL based on Pearson Correlation using complete linkage clustering of genes only, with optimization of Gene Leaf Order. Different distance thresholds (0.69-1.14) were then used to separate each of the four KMC Support clusters into 2 or 3 sub-clusters.

#### TE calculation and identification of regulatory mechanism

Median values of read counts for edgeR-normalized mRNA and ribosome footprint read counts were used to represent each gene in each stage. The TE (ratio of ribosome footprint to mRNA read counts) for each gene was calculated for each sample and the median TE of those values for each condition was used. Gene-level fold changes between biological conditions for TE, mRNA and ribosome footprint and their log_2_ ratios calculated.

We used DESeq [49] to apply GLMs to the raw count data from CDS regions (of both fragmented-mRNA and ribosome profiling experiments). The four generalized linear models used corresponding to the following potential regulatory mechanisms: a) mRNA abundance change only, b) TE change only, c) full model (both mRNA abundance and TE change), and d) no (significant) regulation. The raw count data from each gene was tested for its fit to each model and a deviation score was calculated given the average read counts. If the fit to the full model was better than the no-regulation model (with statistical support of FDR < 0.01), the gene was further analyzed. To be categorized as regulated by mRNA abundance, the following three criteria had to be met with statistical support: 1) the fit to the mRNA model was statistically significantly (FDR < 0.01) better than to the no-regulation model; 2) the TE model was not significantly better than the no-regulation model; and 3) the full model was not significantly better than mRNA model. The same logic was followed to assign genes to the TE model. The remaining genes were all assigned to the full model.

#### Gene categories and descriptions

Due to the divergence of trypanosomatids from humans and model organisms, many genes lack GO terms or are mis-categorized. Therefore, we manually updated our previous functional categorization of genes into molecular categories [7] and, when possible, subcellular categories. This was accomplished by reviewing the gene descriptions and user comments of all genes on TriTrypDB, as well as examination of the literature. In some cases, the presence of InterPro domains on “hypothetical” proteins allowed presumptive functional categorization.

#### uORFs

We developed an *ad-hoc* PERL script, which contains the get_orf algorithm from the EMBOSS package [72], to search for uORFs. No minimum size was specified. The region between the major SL addition site and the main CDS start codon was scanned for additional ATG start codons and, if one was encountered, the uORF was extended 3′ to the next in-frame stop codon. For assignment of reads, the entire uORF was utilized. RRSs were calculated as above, except that reads were counted in the uORF if they were fully contained in the region from nt -12 (relative to the ATG) to 6 nt past the stop codon. Reads were included in the 3′ UTR counts if they were fully contained in the region from the stop codon until 13 nt before the main CDS start codon or until the next ATG in any reading frame, whichever came first.

## Electronic supplementary material

Additional file 1: Table S1: Biological samples. **Table S2.** Library statistics. **Table S3.** Median reads per kb across coding regions and UTRs from representative libraries. **Table S4.** Mechanism of stage-specific regulation of protein production, by gene category. **Table S5.** Primers. (DOCX 47 KB)

Additional file 2: Figure S1: Experiments related to setup of system and 3 nt periodicity of ribosome footprints. **Figure S2.** A) Technical and biological replicates B) Distribution of read counts in representative mRNA and ribosome profiling libraries. **Figure S3.** Correlations between raw read count values of individual samples. **Figure S4.** Relative read counts around start and stop codons. **Figure S5.** Lack of ribosome profiling signal in 3′ UTRs known to interact with RNA binding proteins. **Figure S6.** Polysome analysis for selected genes with differing TEs. **Figure S7.** High level clustering analysis of ribosome profiling samples. **Figure S8.** Enrichment of functional categories in different gene clusters. **Figure S9.** Enrichment of functional categories regulated by different mechanisms. **Figure S10.** More frequent regulation of genes with high protein production. **Figure S11.** Genes with uORFs show similar regulation to all genes. **Figure S12.** Comparison of our data with Vasquez [20]. **Figure S13.** Correlation between changes in translation and protein abundance. **Figure S14.** Length and composition of 5′ UTRs of ribosomal genes compared to rest of the genome. (PPTX 5 MB)

Additional file 3: **Coordinates, read count data, and RRSs for all CDSs.** (XLSX 4 MB)

Additional file 4: **Gene-level comparison of ribosome footprint, mRNA, and TE across biological samples.** (XLSX 3 MB)

Additional file 5: **Genes with uORFs, uORF read counts, and uORF RRSs.** (XLSX 4 MB)

Additional file 6: **CDS 5′ UTR mapping.** (XLSX 757 KB)

Additional file 7: **CDS start codons used in this study compared to TriTrypDB and other data.** (XLSX 465 KB)

Additional file 8: **Genes removed from analyses with rationale and RRSs.** (XLSX 70 KB)

## Abbreviations

BF: Bloodstream forms
cBF: Cultured bloodstream forms
CDS: Coding sequence
ESAG: Expression site-associated gene
FDR: False discovery rate
GLM: Generalized linear models
PCF: Procyclic cultured forms
RPK: Reads per kB
SL: Spliced leader
slBF: Slender bloodstream forms
uORF: Upstream open reading frame
TE: Calculated translation efficiency
VR: Variant surface glycoprotein-related
VSG: Variant surface glycoprotein.
