# Supplementary material for: Extensive stage-regulation of translation revealed by ribosome profiling of Trypanosoma brucei
Source: BMC Genomics. 2014 Oct 20;15(1):911. doi: 10.1186/1471-2164-15-911 (PMC4210626; doi:10.1186/1471-2164-15-911)
Supplement: Supplementary file 1 — Additional file 1: Table S1: Biological samples. Table S2. Library statistics. Table S3. Median reads per kb across coding regions and UTRs from representative libraries. Table S4. Mechanism of stage-specific regulation of protein production, by gene category. Table S5. Primers. (DOCX 47 KB) [file 12864_2014_6600_MOESM1_ESM.docx]

**Additional File 1, Supplementary Tables, Jensen et al.**

**Table S1. Biological samples**

| ***In vitro* samples** |  | **Cell density** | **24h Fold change** | **Library** | | **Library type** |
| --- | --- | --- | --- | --- | --- | --- |
| PCF1 |  | 1.2×10^7^ | 6 | BJ005,006 | | Ribosome |
|  |  |  |  | BJ008 | | mRNA |
|  |  |  |  |  | |  |
|  |  |  |  | BJ012 | | mRNA |
| PCF3 |  | 5.5×10^6^ | 3.2 | BJ015 | | Ribosome |
|  |  |  |  | BJ016 | | mRNA |
| cBF1 |  | 8×10^5^ | 2 | BJ013 | | Ribosome |
|  |  |  |  | BJ014 | | mRNA |
| cBF2 |  | 1.2×10^6^ | 11 | BJ017 | | Ribosome |
|  |  |  |  | BJ018 | | mRNA |
| cBF3 |  | 1.6×10^6^ | 12.3 | BJ019 | | Ribosome |
|  |  |  |  | BJ020 | | mRNA |
|  |  |  |  |  |  | |
| ***In vivo* samples** | **Stabilate** | **Parasitemias^a^** | **% Slender^b^** | **Library** | **Type** | |
| slBF1 | 308+309 | 3.7×10^7^ | 100 |  |  | |
|  |  | 4.7×10^7^ | 100 | BJ023 | Ribosome | |
|  |  | 5.2×10^7^ | 100 | BJ024 | mRNA | |
| slBF2 | 299 | 1.06×10^8^ | 98.1 | BJ029 | Ribosome | |
|  |  | 7.4×10^7^ | 100 | BJ030 | mRNA | |
| slBF3 | 297-1 | 5.0×10^7^ | 100 |  |  | |
|  |  | 1.01×10^8^ | 100 | BJ031 | Ribosome | |
|  |  | 1.21×10^8^ | 98.3 | BJ032 | mRNA | |

**^a^**Parasites from 2-3 rats were pooled and the pool was divided into aliquots for ribosome profiling and mRNA library preparation

**^b^**Morphologically slender upon microscopic examination

**Table S2. Library statistics**

| **Library #** | **Biol**  **Sample** | **Library type** | **Library size** | **Mapped reads** | **structural gene reads** | **Protein coding + flanking regions^a^** | **Unique reads** |
| --- | --- | --- | --- | --- | --- | --- | --- |
| BJ008 | PCF1 | mRNA | 99,555,799 | 60,449,756 | 56,108,125 | 4,341,631 | 6,660,396 |
| BJ012 | PCF2 | mRNA | 92,938,992 | 39,694,793 | 25,919,227 | 13,775,566 | 9,734,009 |
| BJ016 | PCF3 | mRNA | 62,687,865 | 22,962,494 | 15,056,486 | 7,906,008 | 5,750,923 |
| BJ024 | slBF1 | mRNA | 59,694,251 | 48,516,880 | 19,972,022 | 28,544,858 | 20,057,550 |
| BJ030 | slBF2 | mRNA | 56,468,981 | 48,237,788 | 42,594,168 | 5,643,620 | 4,141,000 |
| BJ032 | slBF3 | mRNA | 61,128,710 | 52,725,108 | 39,960,842 | 12,764,266 | 9,332,518 |
| BJ013 | cBF1 | mRNA | 79,920,340 | 64,727,713 | 33,254,633 | 31,473,080 | 22,684,899 |
| BJ018 | cBF2 | mRNA | 42,244,569 | 23,100,195 | 18,146,868 | 4,953,327 | 4,058,512 |
| BJ020 | cBF3 | mRNA | 101,716,337 | 49,335,934 | 23,844,045 | 25,491,889 | 18,460,966 |
| BJ006 | PCF1 | Ribo | 98,760,826 | 72,852,728 | 64,645,472 | 8,207,256 | 6,812,405 |
| BJ010 | PCF2 | Ribo | 86,223,360 | 40,237,155 | 30,768,765 | 9,468,390 | 6,694,133 |
| BJ015 | PCF3 | Ribo | 64,636,189 | 23,590,420 | 16,654,206 | 6,936,214 | 4,694,032 |
| BJ023 | slBF1 | Ribo | 68,616,219 | 57,998,461 | 44,707,386 | 13,291,075 | 9,330,511 |
| BJ029 | slBF2 | Ribo | 45,749,193 | 19,548,984 | 11,876,731 | 7,672,253 | 6,472,497 |
| BJ031 | slBF3 | Ribo | 48,551,383 | 25,742,265 | 13,554,643 | 12,187,622 | 9,818,698 |
| BJ014 | cBF1 | Ribo | 86,171,690 | 52,145,085 | 46,732,470 | 5,412,615 | 14,192,047 |
| BJ017 | cBF2 | Ribo | 38,236,277 | 15,046,999 | 5,838,882 | 9,208,117 | 6,242,902 |
| BJ019 | cBF3 | Ribo | 69,724,475 | 15,232,597 | 9,026,205 | 6,206,392 | 4,575,340 |

^a^ exclusive of structural RNA genes

**Table S3. Median reads per kb across coding regions and UTRs from representative libraries^a^**

| **Sample** | **Library** | **5’UTR** | **CDS** | **3’UTR** |
| --- | --- | --- | --- | --- |
| PCF3 | Ribosome | 93 | 194 | 3 |
| PCF3 | mRNA | 219 | 320 | 243 |
| slBF1 | Ribosome | 320 | 412 | 5 |
| slBF1 | mRNA | 1036 | 1220 | 1074 |

^a^Reads per kb were calculated for the 5700+ genes with defined UTRs. The 5’ UTR and CDS were as outlined in the Methods. The 3’ UTR was taken from TriTrypDB and reads fully contained in that region were included in these counts.

**Table S4. Mechanism of stage-specific regulation of protein production, by gene category**

| **Stage up-regulated** | **PCF** | | | **slBF** | | |  |
| --- | --- | --- | --- | --- | --- | --- | --- |
| **Category** | **mRNA** | **both** | **TE** | **mRNA** | **both** | **TE** | **Total** |
| DNA-associated | 9 | 24 | 1 | 0 | 9 | 1 | **44** |
| ESAG/GRESAG | 0 | 2 | 2 | 45 | 44 | 0 | **93** |
| Interacting | 3 | 11 | 0 | 13 | 30 | 5 | **62** |
| Metabolism | 127 | 85 | 6 | 19 | 47 | 8 | **292** |
| Organelle biogenesis/structure | 11 | 24 | 6 | 9 | 24 | 1 | **75** |
| Other | 33 | 34 | 0 | 9 | 46 | 2 | **124** |
| phosphorylation | 3 | 17 | 2 | 30 | 48 | 6 | **106** |
| Protease-related | 13 | 14 | 2 | 6 | 37 | 3 | **75** |
| Protein folding | 35 | 17 |  | 2 | 12 | 1 | **67** |
| Protein transport/modification | 4 | 10 | 1 | 10 | 50 | 10 | **85** |
| RNA-associated | 15 | 46 | 4 | 8 | 13 | 3 | **89** |
| Transcription | 1 | 1 |  |  | 3 |  | **5** |
| Translation | 79 | 158 | 4 |  | 4 |  | **245** |
| Transporter | 20 | 21 | 3 | 18 | 39 | 10 | **111** |
| Unknown: conserved | 119 | 256 | 27 | 129 | 380 | 63 | **974** |
| Unknown: Tb-specific | 13 | 26 | 1 | 30 | 56 | 13 | **139** |
| VSG/VR | 0 | 0 | 0 | 35 | 28 | 0 | **63** |
| **Total** | **485** | **746** | **59** | **363** | **870** | **126** | **2649** |

^a^ Gene counts are listed for those up-regulated in PCF or slBF. Yellow highlights categories that are more discussed in the text.

**Table S5. Primers**

| **Primer** | **Sequence 5’-3’** | | **Purpose^a^** | |  |
| --- | --- | --- | --- | --- | --- |
| RP_index_RT | Phos-AGATCGGAAGAGCGTCGTGTAGGGAAAGAGTGTAGATCTCGGTGGTCGC-spacer 18-CACTCA-spacer 18-TTCAGACGTGTGCTCTTCCGATCTATTGATGGTGCCTACAG | | Reverse  transcription | |  |
| RP_index_PCR  forward | AATGATACGGCGACCACCGAGATCTACAC | | RP PCR forward | |  |
| RP_index_PCR  rev_1 | CAAGCAGAAGACGGCATACGAGATAGTCGTGTGACTGGAGTTCAGACGTGTGCTCTTCCG | | RP and SL PCR reverse | |  |
| RP_index_PCR  rev_2 | CAAGCAGAAGACGGCATACGAGATACTGATGTGACTGGAGTTCAGACGTGTGCTCTTCCG | | RP and SL PCR reverse | |  |
| RP_index_PCR  rev_3 | CAAGCAGAAGACGGCATACGAGATATGCTGGTGACTGGAGTTCAGACGTGTGCTCTTCCG | | RP and SL PCR reverse | |  |
| RP_index_PCR  rev_4 | CAAGCAGAAGACGGCATACGAGATACGTCGGTGACTGGAGTTCAGACGTGTGCTCTTCCG | | RP and SL PCR reverse | |  |
| RP_index_PCR  rev_5 | CAAGCAGAAGACGGCATACGAGATAGCTGCGTGACTGGAGTTCAGACGTGTGCTCTTCCG | | RP and SL PCR reverse | |  |
| Tb sub 18S | Biotin-ACGGGAATATCCTCAGCACGTTTCTT | | subtraction | |  |
| Tb sub LSU 1-1 | Biotin-TAGCCACGGAGCCTACGCGCCTCCTCCT | | subtraction | |  |
| Tb sub LSU 1-2 | Biotin-TTGCAAAGCAAACCCGTTGCTGAACAC | | subtraction | |  |
| Tb sub LSU 1-3 | Biotin-TAGGGCTGGTGCAGGCGTGGCGGAATTC | | subtraction | |  |
| Tb sub LSU 1-4 | Biotin-TTTACAACCCTTCATGTGAGTATTGAGCC | | subtraction | |  |
| Tb sub LSU 2 | Biotin-TGCGCAGTCTTCGGGCTGTGCGCCGTCTAGG | | subtraction | |  |
| Random5 | ACTGGAGTTCAGACGTGTGCTCTTCCGATCTNNNNNN | | SL library  1^st^ strand | |  |
| SL_2nd primer3 | AGATCTACACTCTTTCCCTACACGACGCTCTTCCGATCTCAGTTTCTGTACT | | SL library  2^nd^ strand | |  |
| Multi-PCR P2 | AATGATACGGCGACCACCGAGATCTACACTCTTTCCCTAC | | SL library  PCR forward | |  |
| SL_SEQ_Primer2 | | CTACACGACGCTCTTCCGATCTCAGTTTCTGTACTTTATTG | | SL sequencing | |

**^a^**RP-ribosome profiling; SL-spliced leader. Indexing sequences for multiplexing are underlined.
