# Supplementary material for: Extensive stage-regulation of translation revealed by ribosome profiling of Trypanosoma brucei
Source: BMC Genomics. 2014 Oct 20;15(1):911. doi: 10.1186/1471-2164-15-911 (PMC4210626; doi:10.1186/1471-2164-15-911)
Supplement: Supplementary file 2 — Additional file 2: Figure S1: Experiments related to setup of system and 3 nt periodicity of ribosome footprints. Figure S2. A) Technical and biological replicates B) Distribution of read counts in representative mRNA and ribosome profiling libraries. Figure S3. Correlations between raw read count values of individual samples. Figure S4. Relative read counts around start and stop codons. Figure S5. Lack of ribosome profiling signal in 3′ UTRs known to interact with RNA binding proteins. Figure S6. Polysome analysis for selected genes with differing TEs. Figure S7. High level clustering analysis of ribosome profiling samples. Figure S8. Enrichment of functional categories in different gene clusters. Figure S9. Enrichment of functional categories regulated by different mechanisms. Figure S10. More frequent regulation of genes with high protein production. Figure S11. Genes with uORFs show similar regulation to all genes. Figure S12. Comparison of our data with Vasquez [20]. Figure S13. Correlation between changes in translation and protein abundance. Figure S14. Length and composition of 5′ UTRs of ribosomal genes compared to rest of the genome. (PPTX 5 MB) [file 12864_2014_6600_MOESM2_ESM.pptx]

## Slide 1
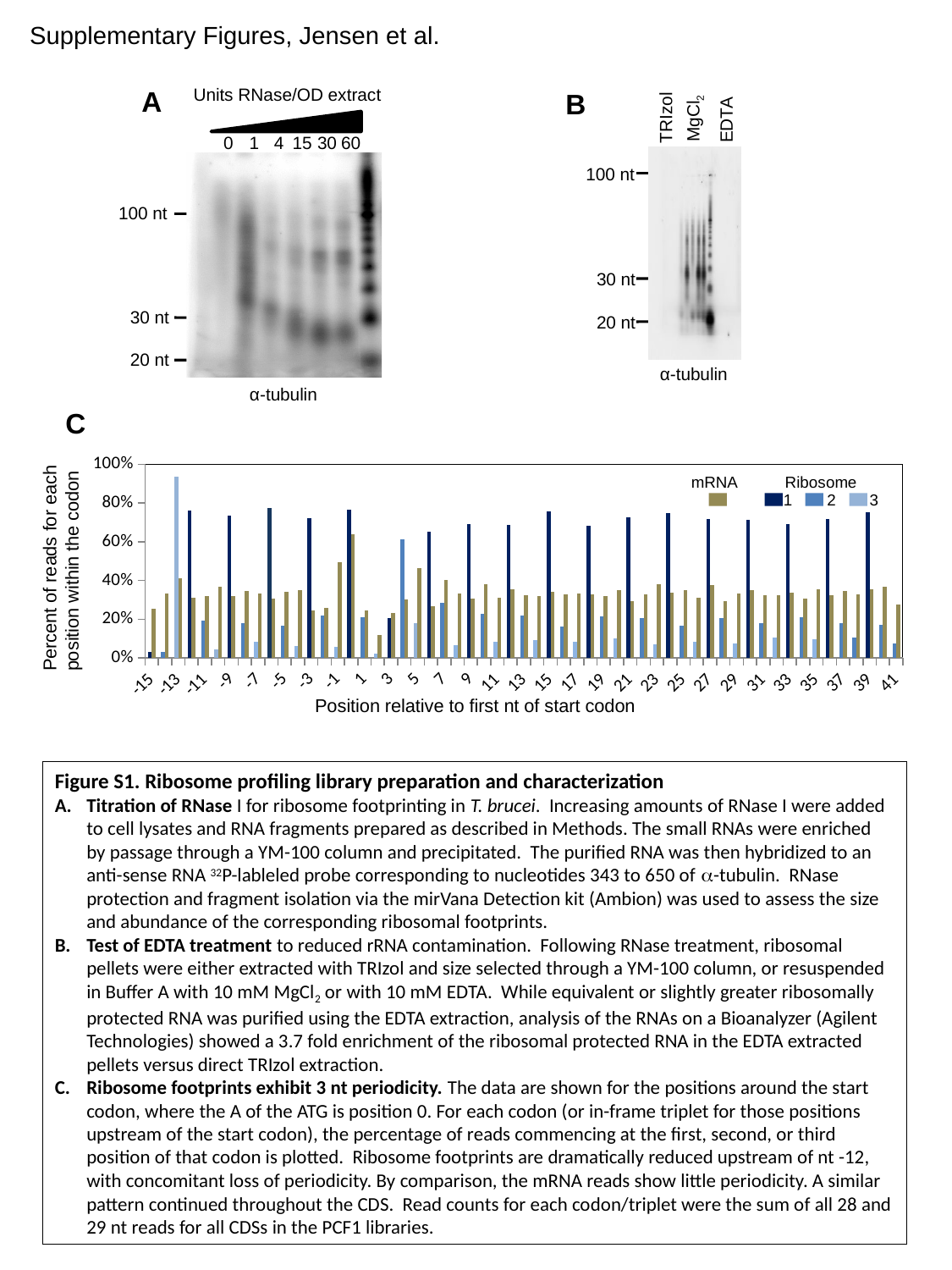

Supplementary Figures, Jensen et al.
B
MgCl2
TRIzol
EDTA
100 nt
30 nt
20 nt
α-tubulin
Units RNase/OD extract
A
0
1
4
15
30
60
100 nt
30 nt
20 nt
α-tubulin
C
### Chart
| Category | Ribosome | mRNA |
|---|---|---|
| -15 | 0.03073624 | 0.253706755 |
| -14 | 0.032999762 | 0.334431631 |
| -13 | 0.936263998 | 0.411861614 |
| -12 | 0.762498916 | 0.30964467 |
| -11 | 0.193580655 | 0.32106599 |
| -10 | 0.043920428 | 0.36928934 |
| -9 | 0.737112847 | 0.318881119 |
| -8 | 0.17845408 | 0.346853147 |
| -7 | 0.084433073 | 0.334265734 |
| -6 | 0.772451271 | 0.307790549 |
| -5 | 0.165719356 | 0.343550447 |
| -4 | 0.061829373 | 0.348659004 |
| -3 | 0.721027383 | 0.247292419 |
| -2 | 0.221107719 | 0.259025271 |
| -1 | 0.057864898 | 0.49368231 |
| 0 | 0.764866756 | 0.637432188 |
| 1 | 0.210392419 | 0.244122966 |
| 2 | 0.024740826 | 0.118444846 |
| 3 | 0.205189962 | 0.23316913 |
| 4 | 0.614559863 | 0.300492611 |
| 5 | 0.180250175 | 0.466338259 |
| 6 | 0.650675051 | 0.266094421 |
| 7 | 0.284520067 | 0.402002861 |
| 8 | 0.064804883 | 0.331902718 |
| 9 | 0.689356601 | 0.305220884 |
| 10 | 0.228364637 | 0.382864793 |
| 11 | 0.082278762 | 0.311914324 |
| 12 | 0.687566258 | 0.354395604 |
| 13 | 0.218032208 | 0.324175824 |
| 14 | 0.094401535 | 0.321428571 |
| 15 | 0.756962933 | 0.340702211 |
| 16 | 0.160608233 | 0.326397919 |
| 17 | 0.082428835 | 0.33289987 |
| 18 | 0.681193908 | 0.328867235 |
| 19 | 0.215528964 | 0.321559074 |
| 20 | 0.103277128 | 0.349573691 |
| 21 | 0.726286849 | 0.292954265 |
| 22 | 0.20497938 | 0.327564895 |
| 23 | 0.068733771 | 0.379480841 |
| 24 | 0.749616938 | 0.336814621 |
| 25 | 0.164995125 | 0.351174935 |
| 26 | 0.085387937 | 0.312010444 |
| 27 | 0.718653521 | 0.375876578 |
| 28 | 0.20525844 | 0.291725105 |
| 29 | 0.076088039 | 0.332398317 |
| 30 | 0.71487691 | 0.348635236 |
| 31 | 0.180390492 | 0.325062035 |
| 32 | 0.104732598 | 0.32630273 |
| 33 | 0.691428571 | 0.339007092 |
| 34 | 0.20952381 | 0.306382979 |
| 35 | 0.099047619 | 0.354609929 |
| 36 | 0.717632552 | 0.323962517 |
| 37 | 0.178032818 | 0.346720214 |
| 38 | 0.10433463 | 0.329317269 |
| 39 | 0.752371638 | 0.355529132 |
| 40 | 0.172518337 | 0.36979786 |
| 41 | 0.075110024 | 0.274673008 |mRNA
Ribosome
1
2
3
Percent of reads for each position within the codon
Position relative to first nt of start codon
Figure S1. Ribosome profiling library preparation and characterization
Titration of RNase I for ribosome footprinting in T. brucei. Increasing amounts of RNase I were added to cell lysates and RNA fragments prepared as described in Methods. The small RNAs were enriched by passage through a YM-100 column and precipitated. The purified RNA was then hybridized to an anti-sense RNA 32P-lableled probe corresponding to nucleotides 343 to 650 of α-tubulin. RNase protection and fragment isolation via the mirVana Detection kit (Ambion) was used to assess the size and abundance of the corresponding ribosomal footprints.
Test of EDTA treatment to reduced rRNA contamination. Following RNase treatment, ribosomal pellets were either extracted with TRIzol and size selected through a YM-100 column, or resuspended in Buffer A with 10 mM MgCl2 or with 10 mM EDTA. While equivalent or slightly greater ribosomally protected RNA was purified using the EDTA extraction, analysis of the RNAs on a Bioanalyzer (Agilent Technologies) showed a 3.7 fold enrichment of the ribosomal protected RNA in the EDTA extracted pellets versus direct TRIzol extraction.
Ribosome footprints exhibit 3 nt periodicity. The data are shown for the positions around the start codon, where the A of the ATG is position 0. For each codon (or in-frame triplet for those positions upstream of the start codon), the percentage of reads commencing at the first, second, or third position of that codon is plotted. Ribosome footprints are dramatically reduced upstream of nt -12, with concomitant loss of periodicity. By comparison, the mRNA reads show little periodicity. A similar pattern continued throughout the CDS. Read counts for each codon/triplet were the sum of all 28 and 29 nt reads for all CDSs in the PCF1 libraries.

## Slide 2
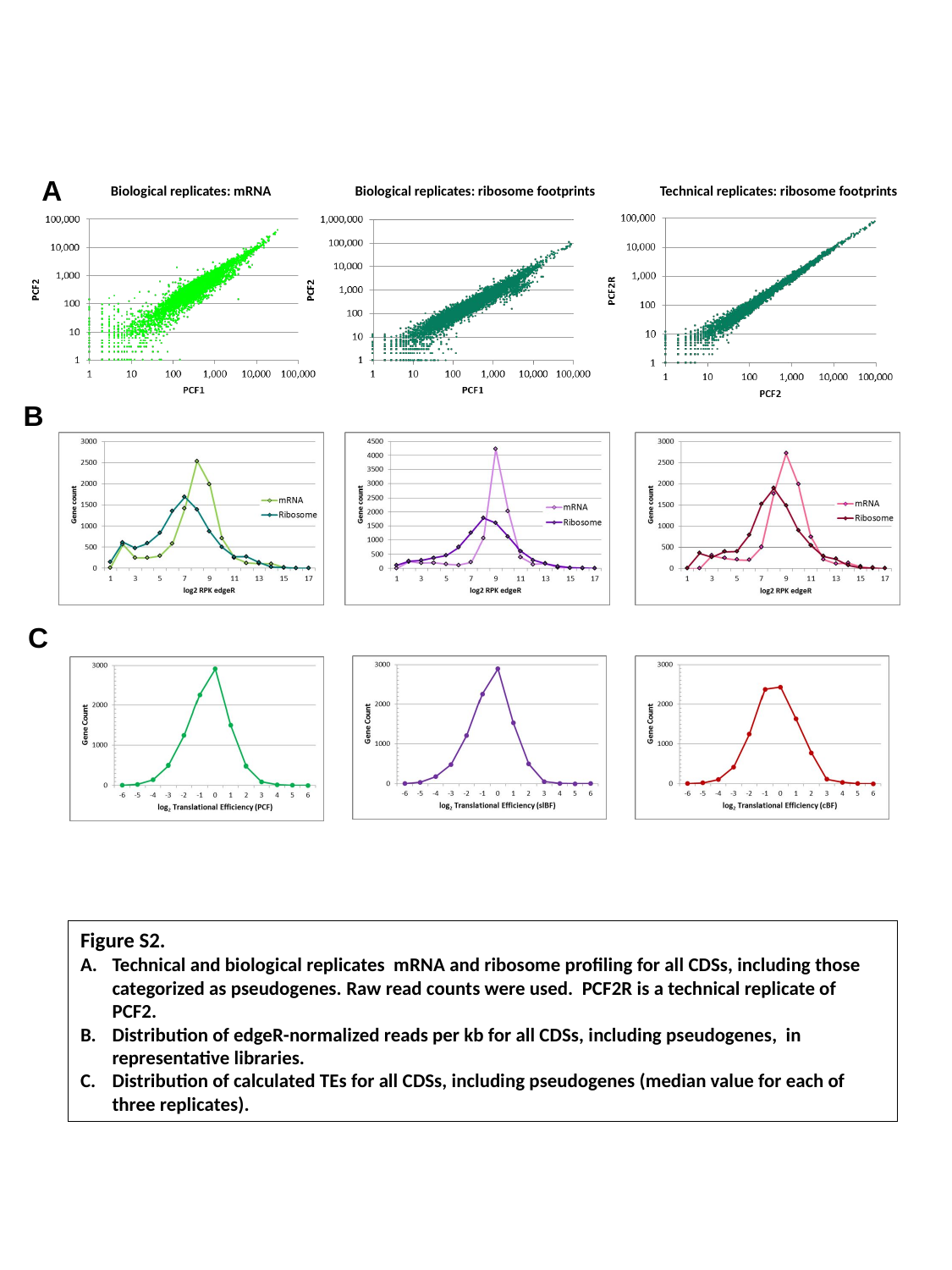

A
Biological replicates: mRNA
Biological replicates: ribosome footprints
Technical replicates: ribosome footprints
B
C
Figure S2.
Technical and biological replicates mRNA and ribosome profiling for all CDSs, including those categorized as pseudogenes. Raw read counts were used. PCF2R is a technical replicate of PCF2.
Distribution of edgeR-normalized reads per kb for all CDSs, including pseudogenes, in representative libraries.
Distribution of calculated TEs for all CDSs, including pseudogenes (median value for each of three replicates).

## Slide 3
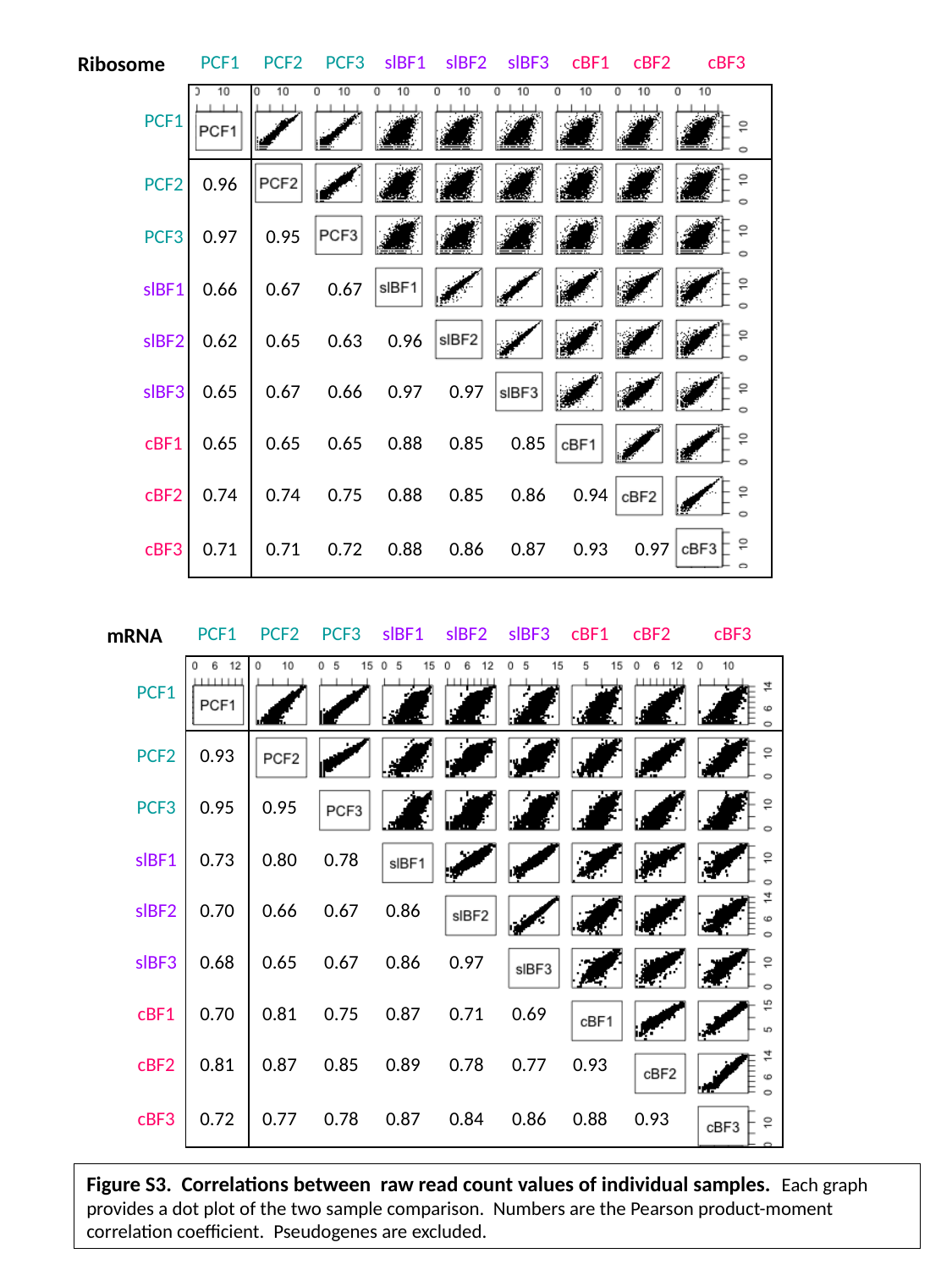

| | PCF1 | PCF2 | PCF3 | slBF1 | slBF2 | slBF3 | cBF1 | cBF2 | cBF3 |
| --- | --- | --- | --- | --- | --- | --- | --- | --- | --- |
| PCF1 | | | | | | | | | |
| PCF2 | 0.96 | | | | | | | | |
| PCF3 | 0.97 | 0.95 | | | | | | | |
| slBF1 | 0.66 | 0.67 | 0.67 | | | | | | |
| slBF2 | 0.62 | 0.65 | 0.63 | 0.96 | | | | | |
| slBF3 | 0.65 | 0.67 | 0.66 | 0.97 | 0.97 | | | | |
| cBF1 | 0.65 | 0.65 | 0.65 | 0.88 | 0.85 | 0.85 | | | |
| cBF2 | 0.74 | 0.74 | 0.75 | 0.88 | 0.85 | 0.86 | 0.94 | | |
| cBF3 | 0.71 | 0.71 | 0.72 | 0.88 | 0.86 | 0.87 | 0.93 | 0.97 | |
Ribosome
| mRNA | PCF1 | PCF2 | PCF3 | slBF1 | slBF2 | slBF3 | cBF1 | cBF2 | cBF3 |
| --- | --- | --- | --- | --- | --- | --- | --- | --- | --- |
| PCF1 | | | | | | | | | |
| PCF2 | 0.93 | | | | | | | | |
| PCF3 | 0.95 | 0.95 | | | | | | | |
| slBF1 | 0.73 | 0.80 | 0.78 | | | | | | |
| slBF2 | 0.70 | 0.66 | 0.67 | 0.86 | | | | | |
| slBF3 | 0.68 | 0.65 | 0.67 | 0.86 | 0.97 | | | | |
| cBF1 | 0.70 | 0.81 | 0.75 | 0.87 | 0.71 | 0.69 | | | |
| cBF2 | 0.81 | 0.87 | 0.85 | 0.89 | 0.78 | 0.77 | 0.93 | | |
| cBF3 | 0.72 | 0.77 | 0.78 | 0.87 | 0.84 | 0.86 | 0.88 | 0.93 | |
mRNA
Figure S3. Correlations between raw read count values of individual samples. Each graph provides a dot plot of the two sample comparison. Numbers are the Pearson product-moment correlation coefficient. Pseudogenes are excluded.

## Slide 4
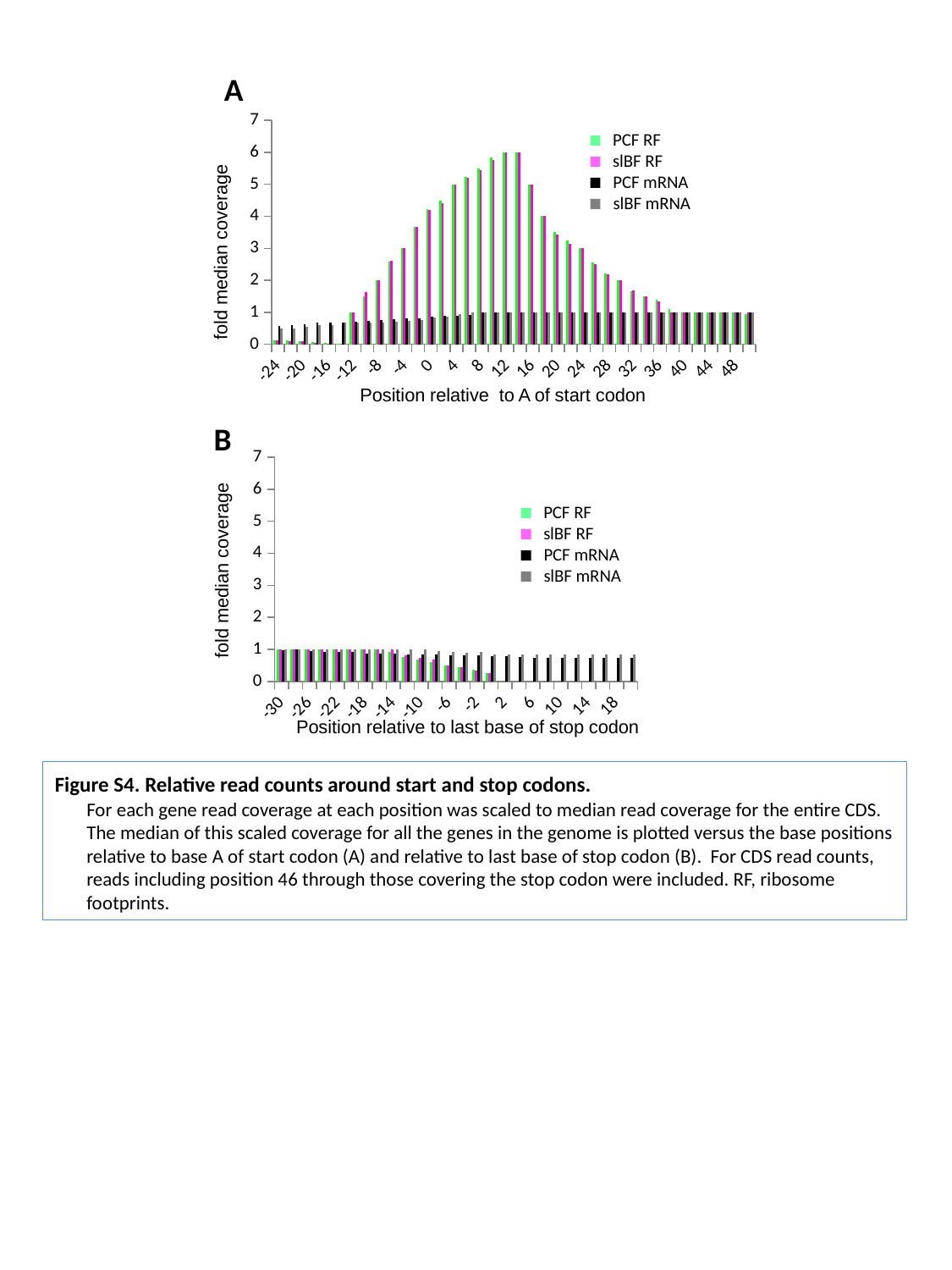

A
### Chart
| Category | PCF.rp | slBF.rp | PCF.mRNA | slBF.mRNA |
|---|---|---|---|---|
| -24 | 0.125 | 0.12258698416368 | 0.575961538461538 | 0.5 |
| -22 | 0.111111111111111 | 0.1 | 0.6 | 0.5 |
| -20 | 0.1 | 0.087049062049062 | 0.625 | 0.545454545454545 |
| -18 | 0.0833333333333333 | 0.0444664031620553 | 0.666666666666667 | 0.6 |
| -16 | 0.0461647727272727 | 0.0145018498942918 | 0.666666666666667 | 0.611111111111111 |
| -14 | 0.017411238679853 | 0.0 | 0.666666666666667 | 0.666666666666667 |
| -12 | 1.0 | 1.0 | 0.7 | 0.666666666666667 |
| -10 | 1.5 | 1.63213530655391 | 0.727272727272727 | 0.666666666666667 |
| -8 | 2.0 | 2.0 | 0.75 | 0.688259109311741 |
| -6 | 2.586965811965809 | 2.62265037593985 | 0.777777777777778 | 0.707142857142857 |
| -4 | 3.0 | 3.0 | 0.8 | 0.732057416267943 |
| -2 | 3.66666666666667 | 3.66666666666667 | 0.8 | 0.75 |
| 0 | 4.209914320685433 | 4.197560975609753 | 0.857142857142857 | 0.833333333333333 |
| 2 | 4.5 | 4.4 | 0.881176470588235 | 0.857142857142857 |
| 4 | 5.0 | 5.0 | 0.9 | 0.943001443001443 |
| 6 | 5.22380952380952 | 5.2 | 0.909090909090909 | 1.0 |
| 8 | 5.5 | 5.44949494949495 | 1.0 | 1.0 |
| 10 | 5.822916666666655 | 5.74709302325581 | 1.0 | 1.0 |
| 12 | 6.0 | 6.0 | 1.0 | 1.0 |
| 14 | 6.0 | 6.0 | 1.0 | 1.0 |
| 16 | 5.0 | 5.0 | 1.0 | 1.0 |
| 18 | 4.0 | 4.0 | 1.0 | 1.0 |
| 20 | 3.5 | 3.42857142857143 | 1.0 | 1.0 |
| 22 | 3.25 | 3.13809523809524 | 1.0 | 1.0 |
| 24 | 3.0 | 3.0 | 1.0 | 1.0 |
| 26 | 2.55061454421304 | 2.5 | 1.0 | 1.0 |
| 28 | 2.2 | 2.174242424242419 | 1.0 | 1.0 |
| 30 | 2.0 | 2.0 | 1.0 | 1.0 |
| 32 | 1.66666666666667 | 1.676944971537 | 1.0 | 1.0 |
| 34 | 1.5 | 1.5 | 1.0 | 1.0 |
| 36 | 1.4 | 1.33333333333333 | 1.0 | 1.0 |
| 38 | 1.11111111111111 | 1.0 | 1.0 | 1.0 |
| 40 | 1.0 | 1.0 | 1.0 | 1.0 |
| 42 | 1.0 | 1.0 | 1.0 | 1.0 |
| 44 | 1.0 | 1.0 | 1.0 | 1.0 |
| 46 | 1.0 | 1.0 | 1.0 | 1.0 |
| 48 | 1.0 | 1.0 | 1.0 | 1.0 |
| 50 | 0.934408602150538 | 1.0 | 1.0 | 1.0 |fold median coverage
Position relative to A of start codon
PCF RF
slBF RF
PCF mRNA
slBF mRNA
B
### Chart
| Category | PCF.rp | slBF.rp | PCF.mRNA | slBF.mRNA |
|---|---|---|---|---|
| -30 | 1.0 | 1.0 | 0.964102564102564 | 1.0 |
| -28 | 1.0 | 1.0 | 1.0 | 1.0 |
| -26 | 1.0 | 1.0 | 0.941176470588235 | 1.0 |
| -24 | 1.0 | 1.0 | 0.928571428571429 | 1.0 |
| -22 | 1.0 | 1.0 | 0.923076923076923 | 1.0 |
| -20 | 1.0 | 1.0 | 0.909090909090909 | 1.0 |
| -18 | 1.0 | 1.0 | 0.875 | 1.0 |
| -16 | 1.0 | 1.0 | 0.857142857142857 | 1.0 |
| -14 | 0.926062593959273 | 1.0 | 0.857142857142857 | 1.0 |
| -12 | 0.75 | 0.8 | 0.833333333333333 | 1.0 |
| -10 | 0.666666666666667 | 0.721111111111111 | 0.833333333333333 | 1.0 |
| -8 | 0.612452107279694 | 0.666666666666667 | 0.833333333333333 | 0.941176470588235 |
| -6 | 0.5 | 0.5 | 0.8 | 0.923076923076923 |
| -4 | 0.436366580310881 | 0.454545454545455 | 0.8 | 0.9 |
| -2 | 0.375 | 0.343303571428571 | 0.8 | 0.924283559577677 |
| 0 | 0.25 | 0.25 | 0.777777777777778 | 0.848076923076923 |
| 2 | 0.0 | 0.0 | 0.777 | 0.849 |
| 4 | 0.0 | 0.0 | 0.755 | 0.848 |
| 6 | 0.0 | 0.0 | 0.743 | 0.841 |
| 8 | 0.0 | 0.0 | 0.742 | 0.843 |
| 10 | 0.0 | 0.0 | 0.741 | 0.845 |
| 12 | 0.0 | 0.0 | 0.74 | 0.847 |
| 14 | 0.0 | 0.0 | 0.739 | 0.848 |
| 16 | 0.0 | 0.0 | 0.739 | 0.839 |
| 18 | 0.0 | 0.0 | 0.738 | 0.838 |
| 20 | 0.0 | 0.0 | 0.7387 | 0.837 |Position relative to last base of stop codon
PCF RF
fold median coverage
slBF RF
PCF mRNA
slBF mRNA
Figure S4. Relative read counts around start and stop codons.
	For each gene read coverage at each position was scaled to median read coverage for the entire CDS. The median of this scaled coverage for all the genes in the genome is plotted versus the base positions relative to base A of start codon (A) and relative to last base of stop codon (B). For CDS read counts, reads including position 46 through those covering the stop codon were included. RF, ribosome footprints.

## Slide 5
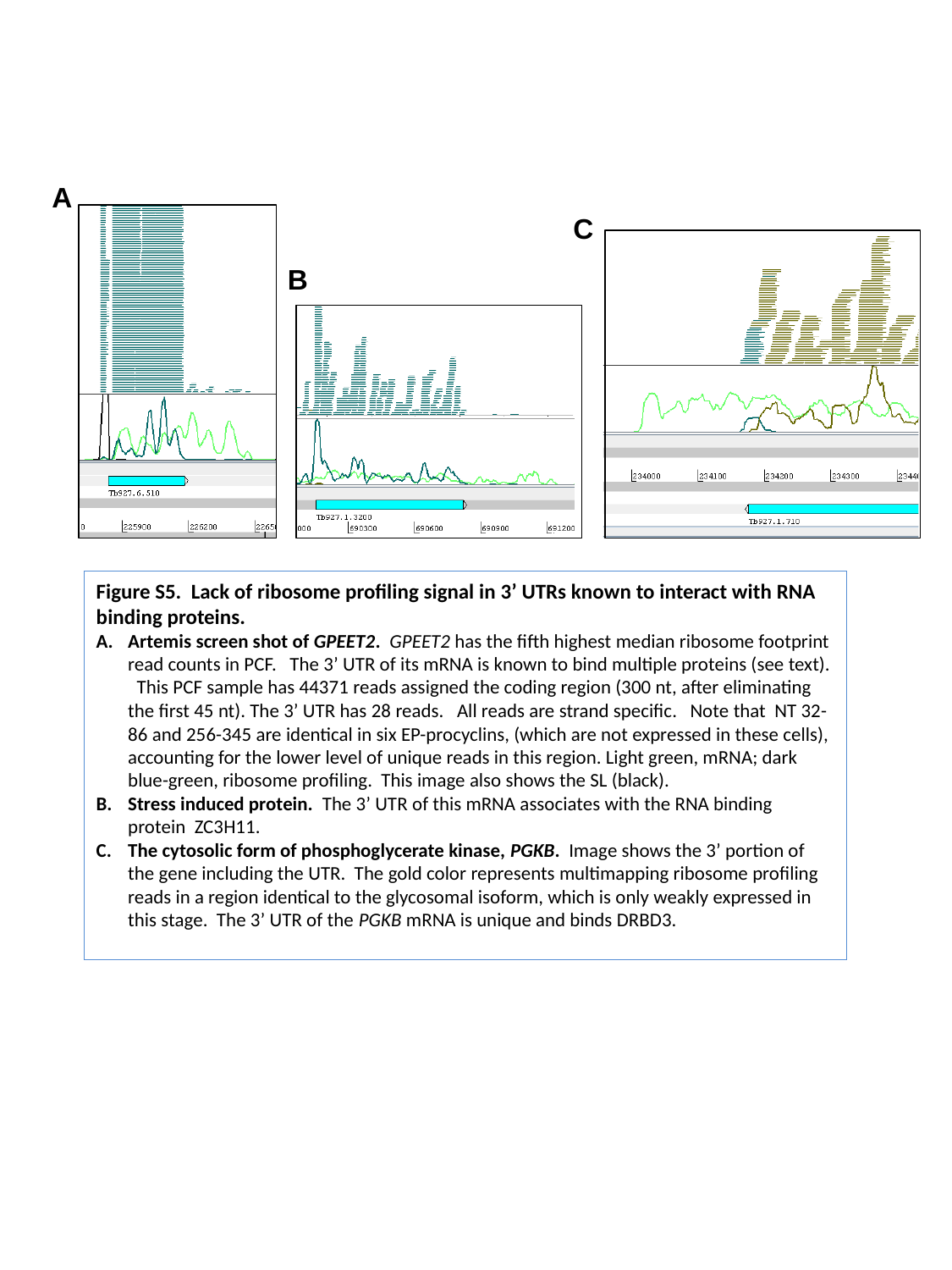

A
C
B
Figure S5. Lack of ribosome profiling signal in 3’ UTRs known to interact with RNA binding proteins.
Artemis screen shot of GPEET2. GPEET2 has the fifth highest median ribosome footprint read counts in PCF. The 3’ UTR of its mRNA is known to bind multiple proteins (see text). This PCF sample has 44371 reads assigned the coding region (300 nt, after eliminating the first 45 nt). The 3’ UTR has 28 reads. All reads are strand specific. Note that NT 32-86 and 256-345 are identical in six EP-procyclins, (which are not expressed in these cells), accounting for the lower level of unique reads in this region. Light green, mRNA; dark blue-green, ribosome profiling. This image also shows the SL (black).
Stress induced protein. The 3’ UTR of this mRNA associates with the RNA binding protein ZC3H11.
The cytosolic form of phosphoglycerate kinase, PGKB. Image shows the 3’ portion of the gene including the UTR. The gold color represents multimapping ribosome profiling reads in a region identical to the glycosomal isoform, which is only weakly expressed in this stage. The 3’ UTR of the PGKB mRNA is unique and binds DRBD3.

## Slide 6
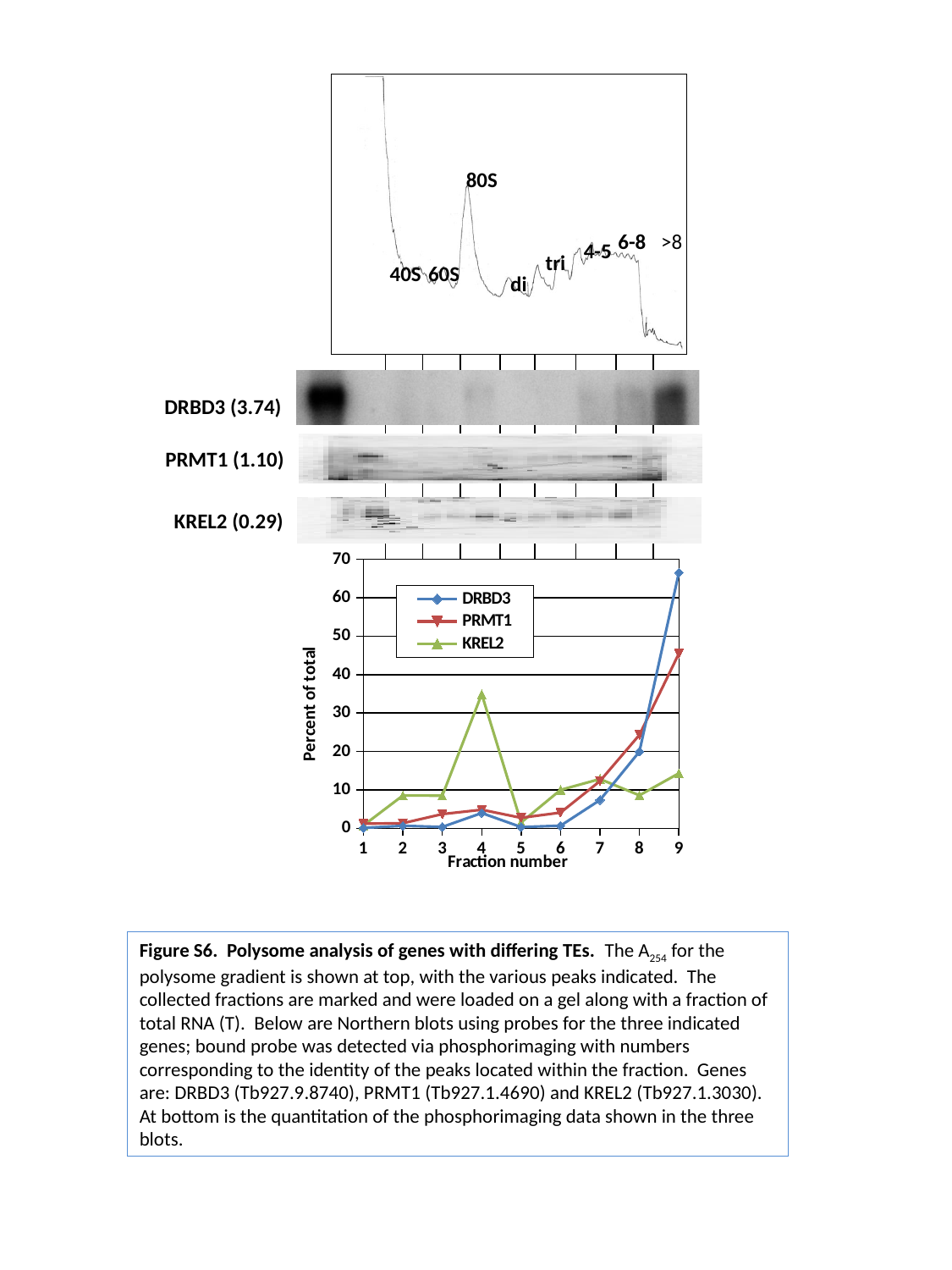

80S
6-8
>8
4-5
tri
40S
60S
di
DRBD3 (3.74)
PRMT1 (1.10)
KREL2 (0.29)
### Chart
| Category | DRBD3 | PRMT1 | KREL2 |
|---|---|---|---|Figure S6. Polysome analysis of genes with differing TEs. The A254 for the polysome gradient is shown at top, with the various peaks indicated. The collected fractions are marked and were loaded on a gel along with a fraction of total RNA (T). Below are Northern blots using probes for the three indicated genes; bound probe was detected via phosphorimaging with numbers corresponding to the identity of the peaks located within the fraction. Genes are: DRBD3 (Tb927.9.8740), PRMT1 (Tb927.1.4690) and KREL2 (Tb927.1.3030). At bottom is the quantitation of the phosphorimaging data shown in the three blots.

## Slide 7
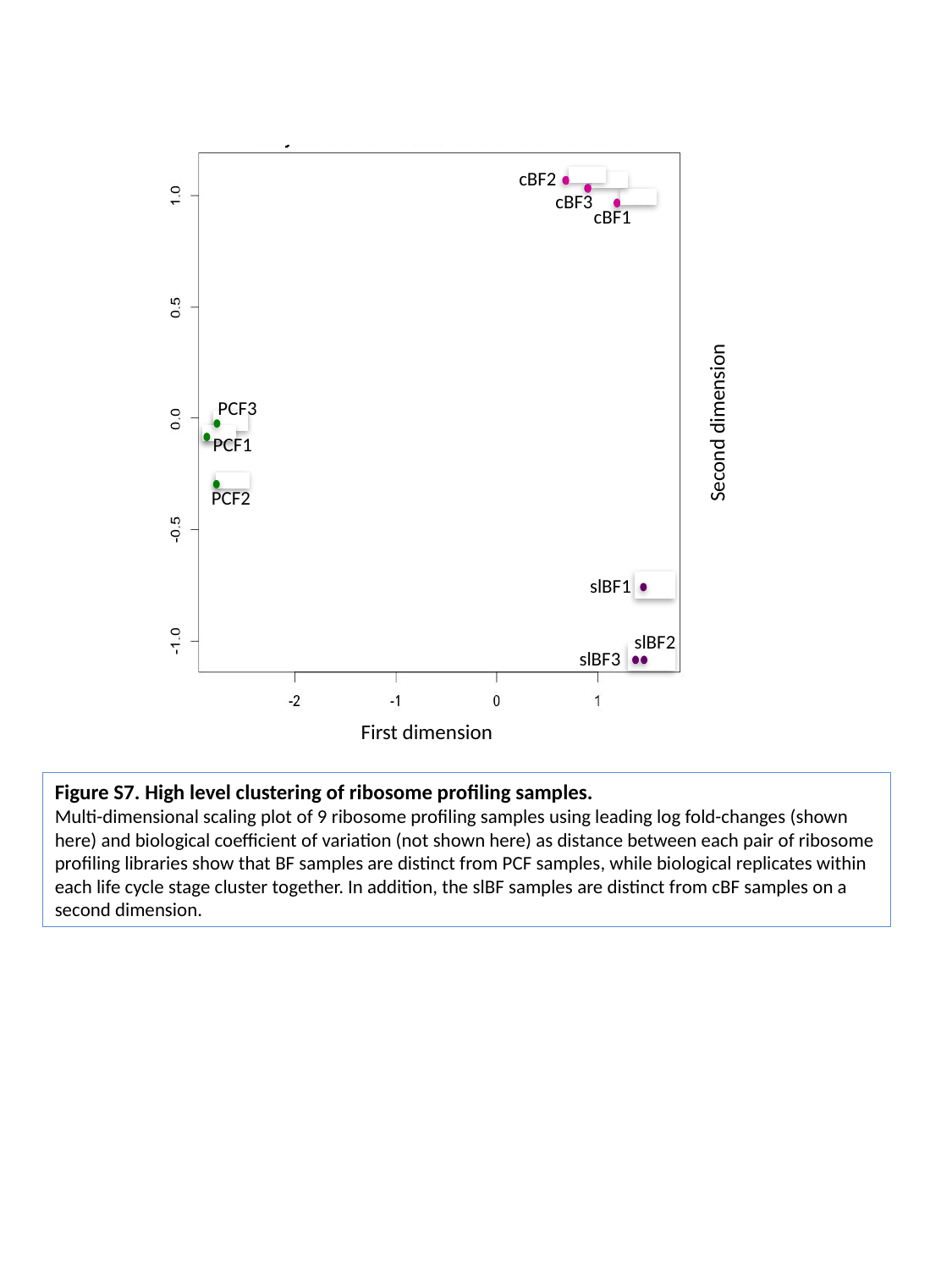

cBF2
cBF3
cBF1
PCF3
Second dimension
PCF1
PCF2
slBF1
slBF2
slBF3
First dimension
Figure S7. High level clustering of ribosome profiling samples.
Multi-dimensional scaling plot of 9 ribosome profiling samples using leading log fold-changes (shown here) and biological coefficient of variation (not shown here) as distance between each pair of ribosome profiling libraries show that BF samples are distinct from PCF samples, while biological replicates within each life cycle stage cluster together. In addition, the slBF samples are distinct from cBF samples on a second dimension.

## Slide 8
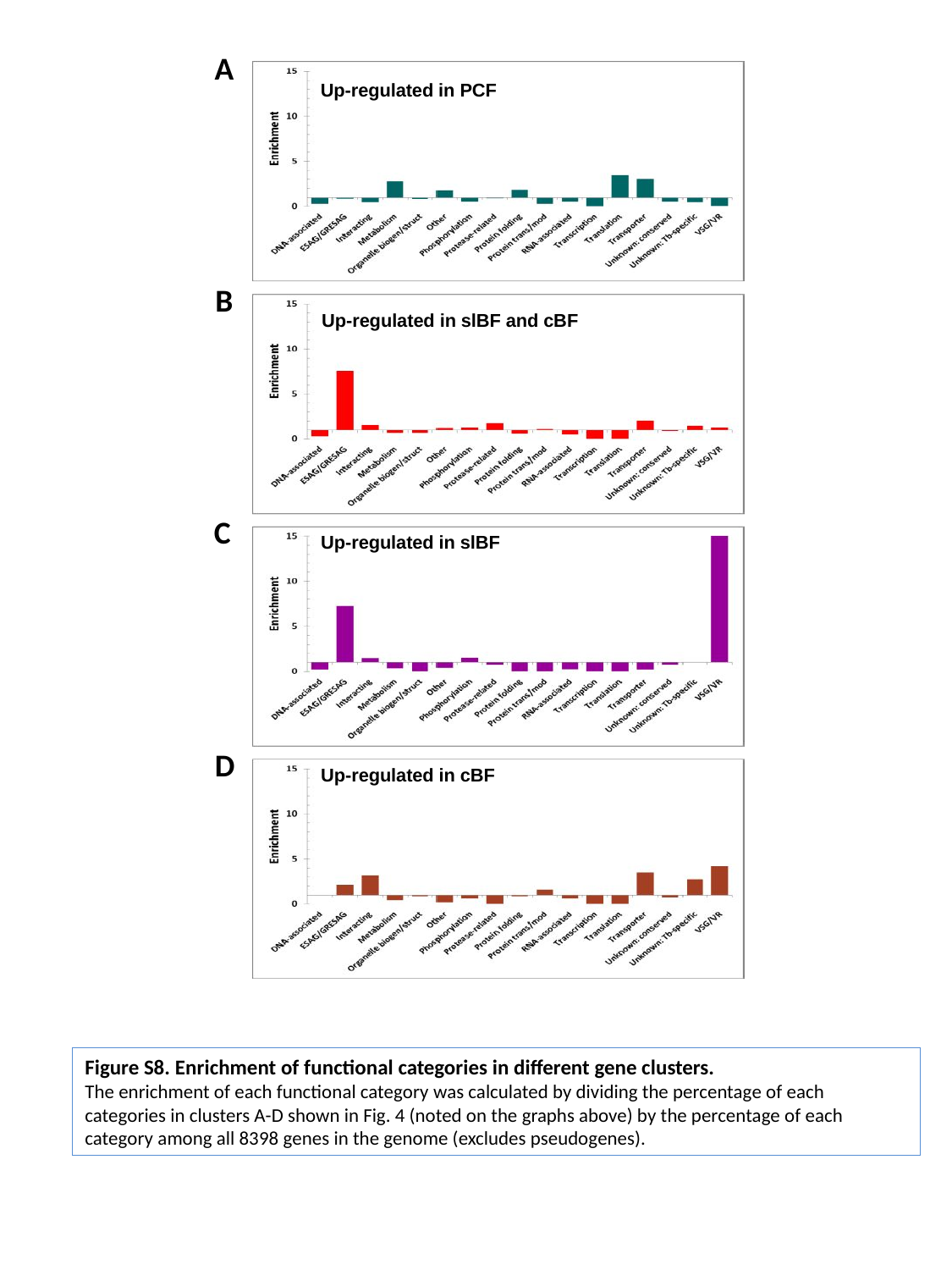

A
B
C
D
Up-regulated in PCF
Up-regulated in slBF and cBF
Up-regulated in slBF
Up-regulated in cBF
Figure S8. Enrichment of functional categories in different gene clusters.
The enrichment of each functional category was calculated by dividing the percentage of each categories in clusters A-D shown in Fig. 4 (noted on the graphs above) by the percentage of each category among all 8398 genes in the genome (excludes pseudogenes).

## Slide 9
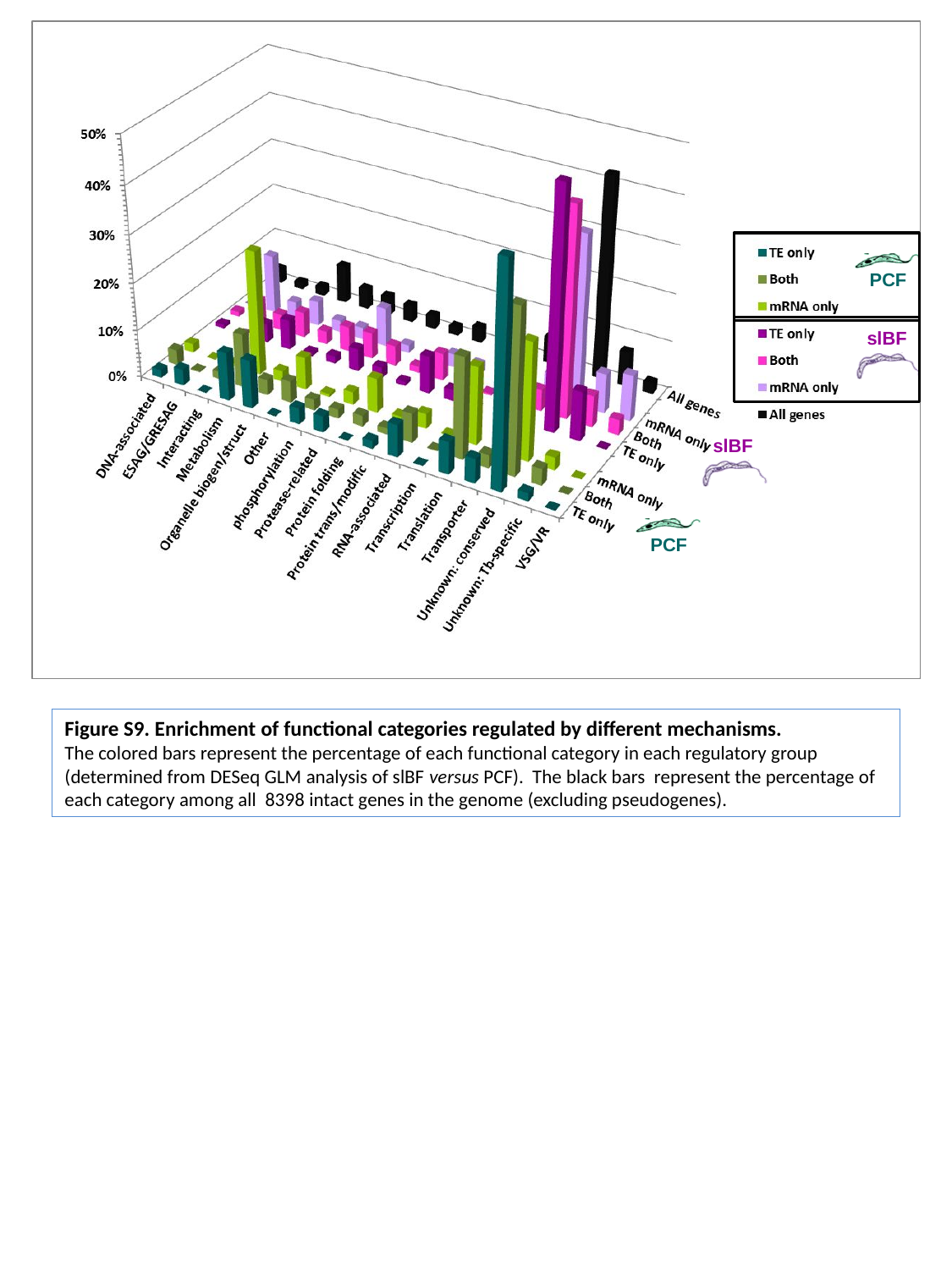

PCF
slBF
slBF
PCF
Figure S9. Enrichment of functional categories regulated by different mechanisms.
The colored bars represent the percentage of each functional category in each regulatory group (determined from DESeq GLM analysis of slBF versus PCF). The black bars represent the percentage of each category among all 8398 intact genes in the genome (excluding pseudogenes).

## Slide 10
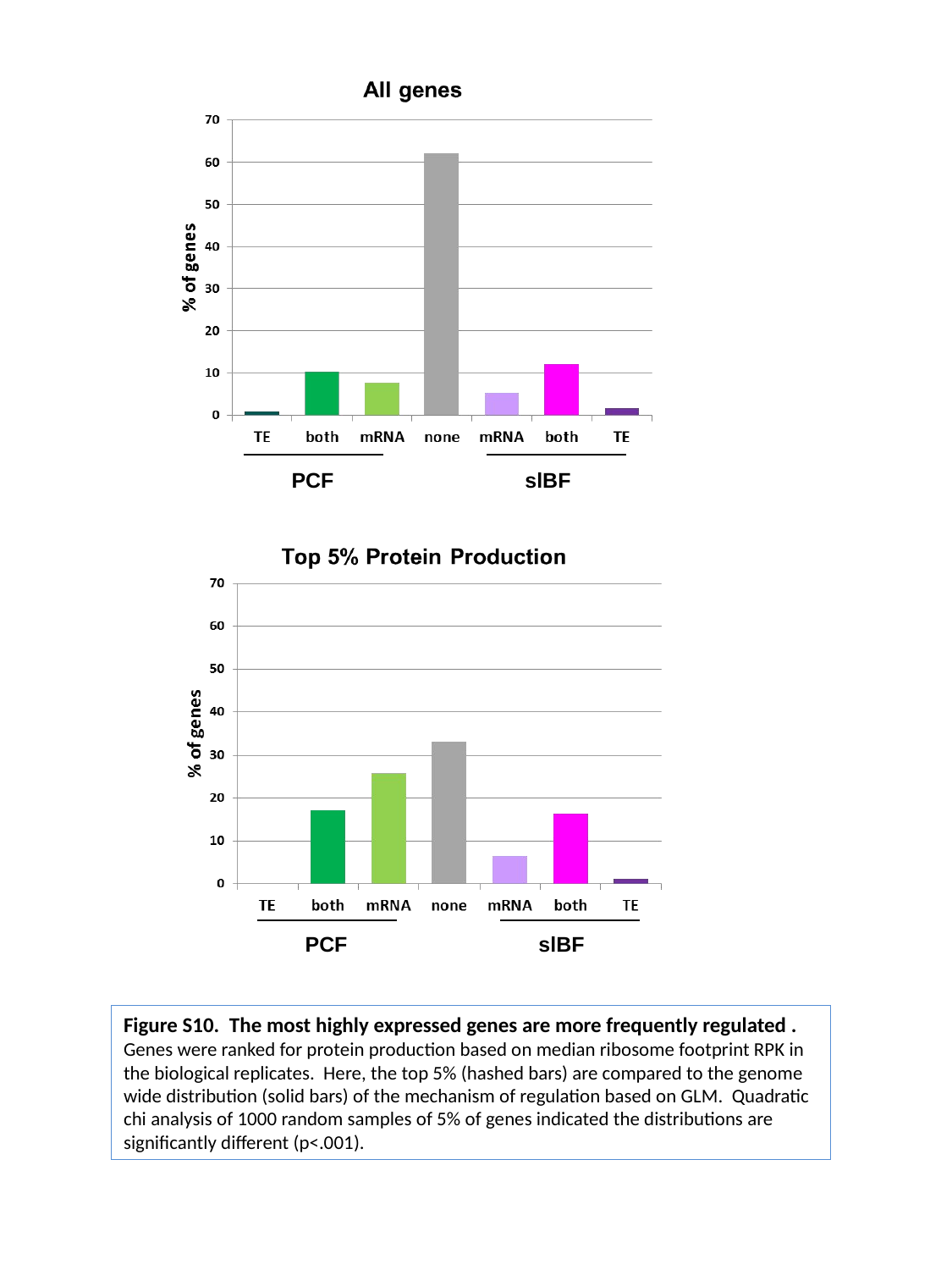

PCF
slBF
PCF
slBF
Figure S10. The most highly expressed genes are more frequently regulated . Genes were ranked for protein production based on median ribosome footprint RPK in the biological replicates. Here, the top 5% (hashed bars) are compared to the genome wide distribution (solid bars) of the mechanism of regulation based on GLM. Quadratic chi analysis of 1000 random samples of 5% of genes indicated the distributions are significantly different (p<.001).

## Slide 11
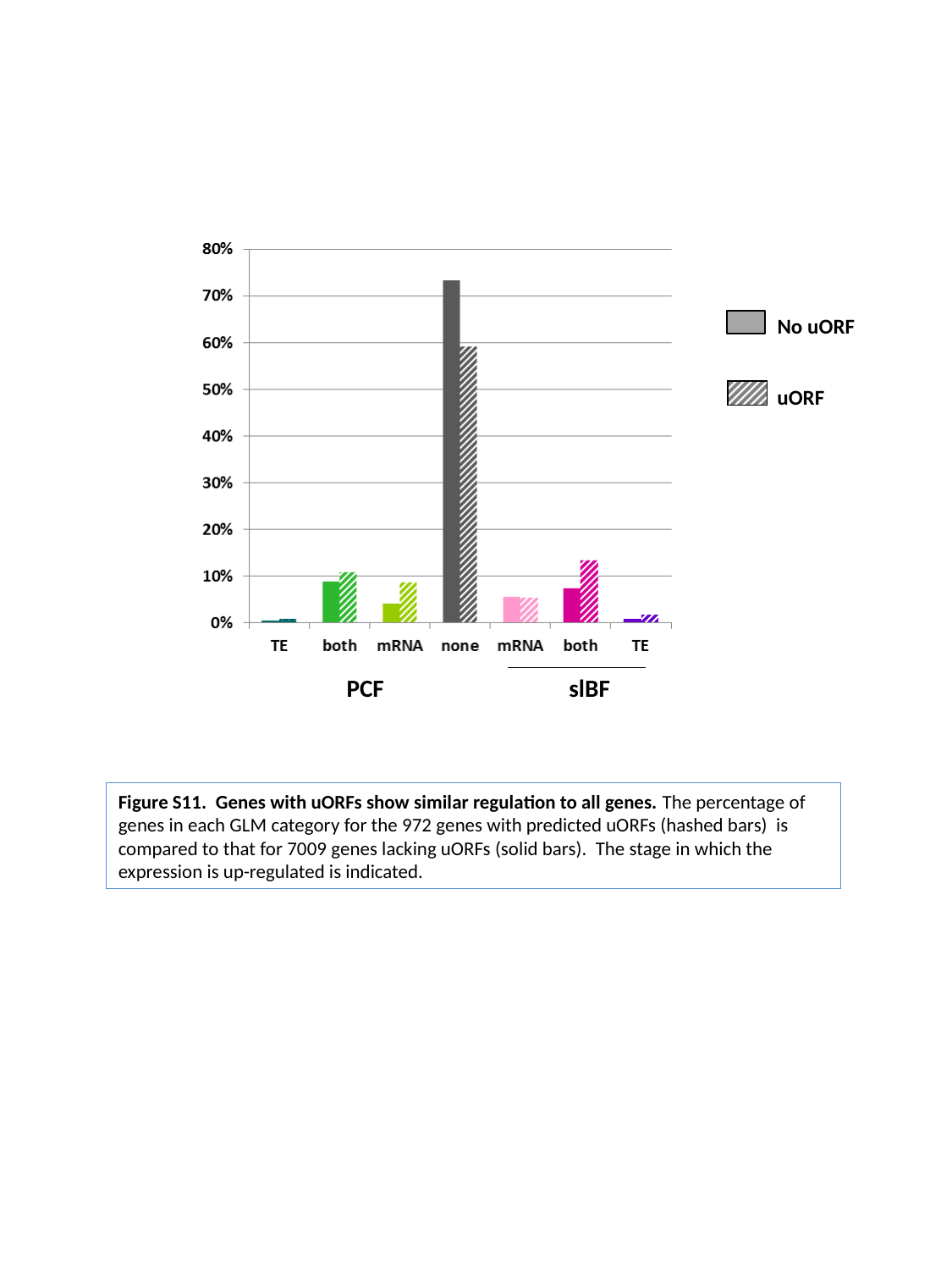

No uORF
uORF
PCF
slBF
Figure S11. Genes with uORFs show similar regulation to all genes. The percentage of genes in each GLM category for the 972 genes with predicted uORFs (hashed bars) is compared to that for 7009 genes lacking uORFs (solid bars). The stage in which the expression is up-regulated is indicated.

## Slide 12
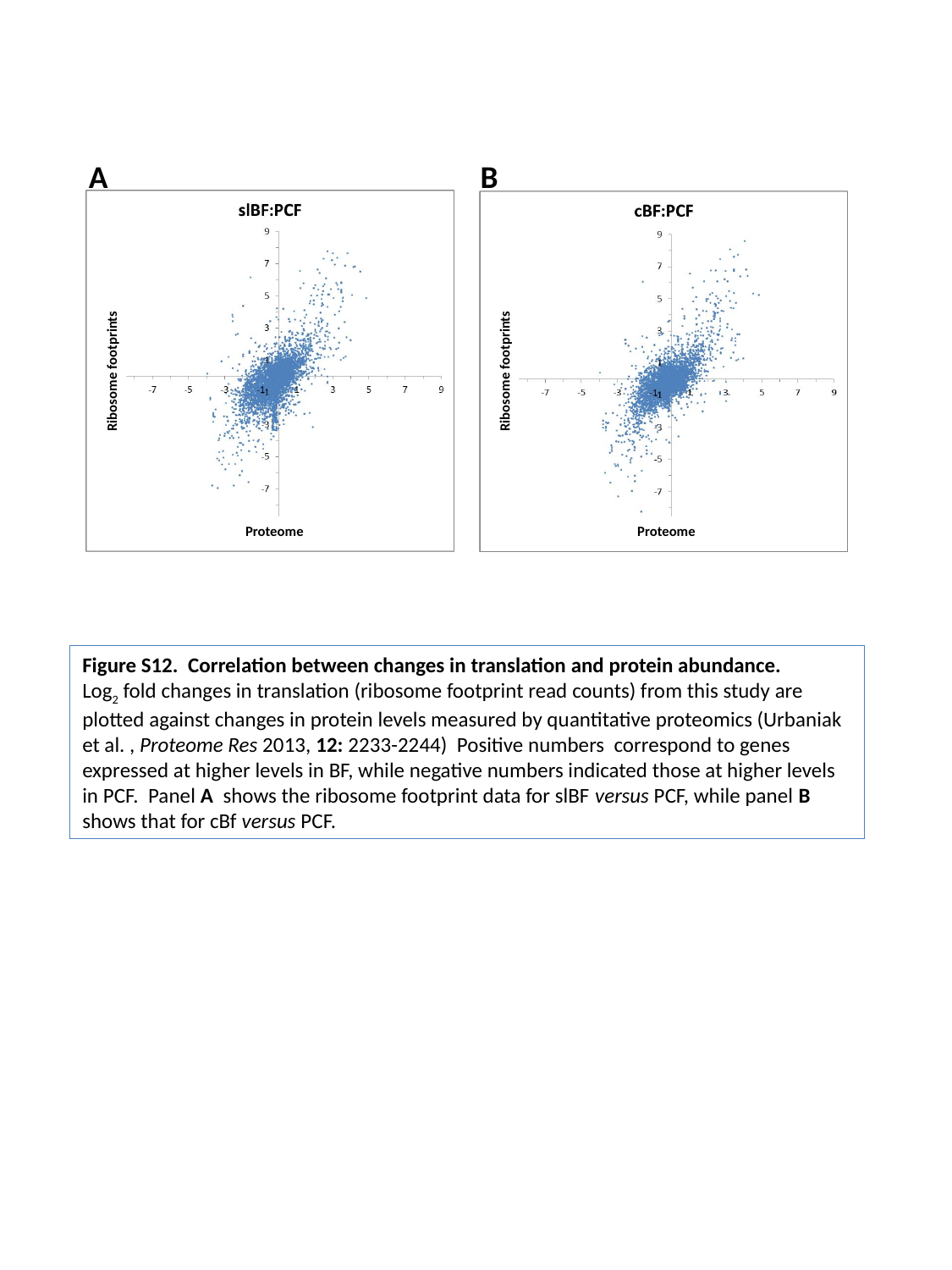

A
B
Ribosome footprints
Ribosome footprints
Proteome
Proteome
Figure S12. Correlation between changes in translation and protein abundance.
Log2 fold changes in translation (ribosome footprint read counts) from this study are plotted against changes in protein levels measured by quantitative proteomics (Urbaniak et al. , Proteome Res 2013, 12: 2233-2244) Positive numbers correspond to genes expressed at higher levels in BF, while negative numbers indicated those at higher levels in PCF. Panel A shows the ribosome footprint data for slBF versus PCF, while panel B shows that for cBf versus PCF.

## Slide 13
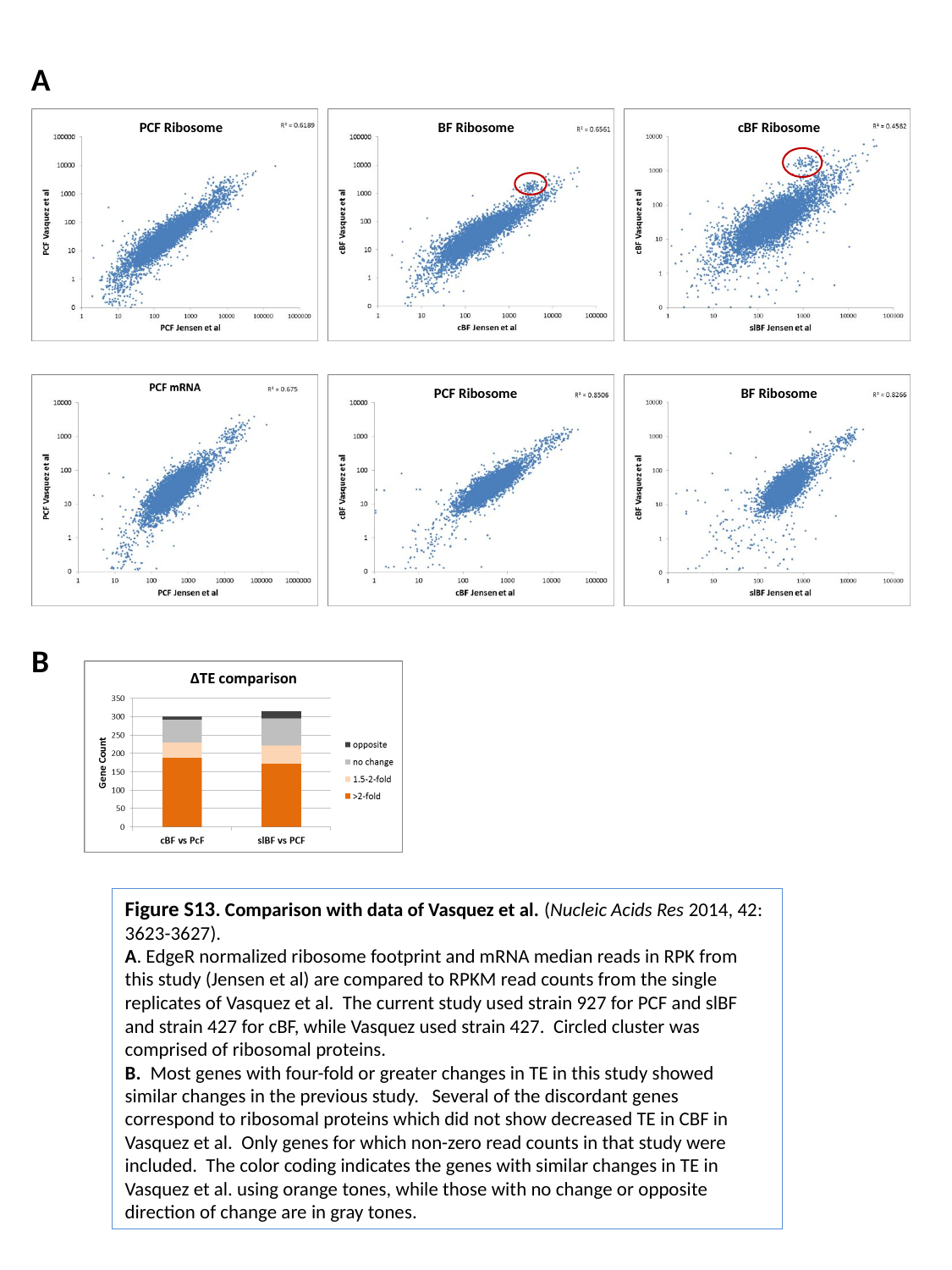

A
PCF Ribosome
BF Ribosome
cBF Ribosome
PCF Ribosome
BF Ribosome
BF Ribosome
B
Figure S13. Comparison with data of Vasquez et al. (Nucleic Acids Res 2014, 42: 3623-3627).
A. EdgeR normalized ribosome footprint and mRNA median reads in RPK from this study (Jensen et al) are compared to RPKM read counts from the single replicates of Vasquez et al. The current study used strain 927 for PCF and slBF and strain 427 for cBF, while Vasquez used strain 427. Circled cluster was comprised of ribosomal proteins.
B. Most genes with four-fold or greater changes in TE in this study showed similar changes in the previous study. Several of the discordant genes correspond to ribosomal proteins which did not show decreased TE in CBF in Vasquez et al. Only genes for which non-zero read counts in that study were included. The color coding indicates the genes with similar changes in TE in Vasquez et al. using orange tones, while those with no change or opposite direction of change are in gray tones.

## Slide 14
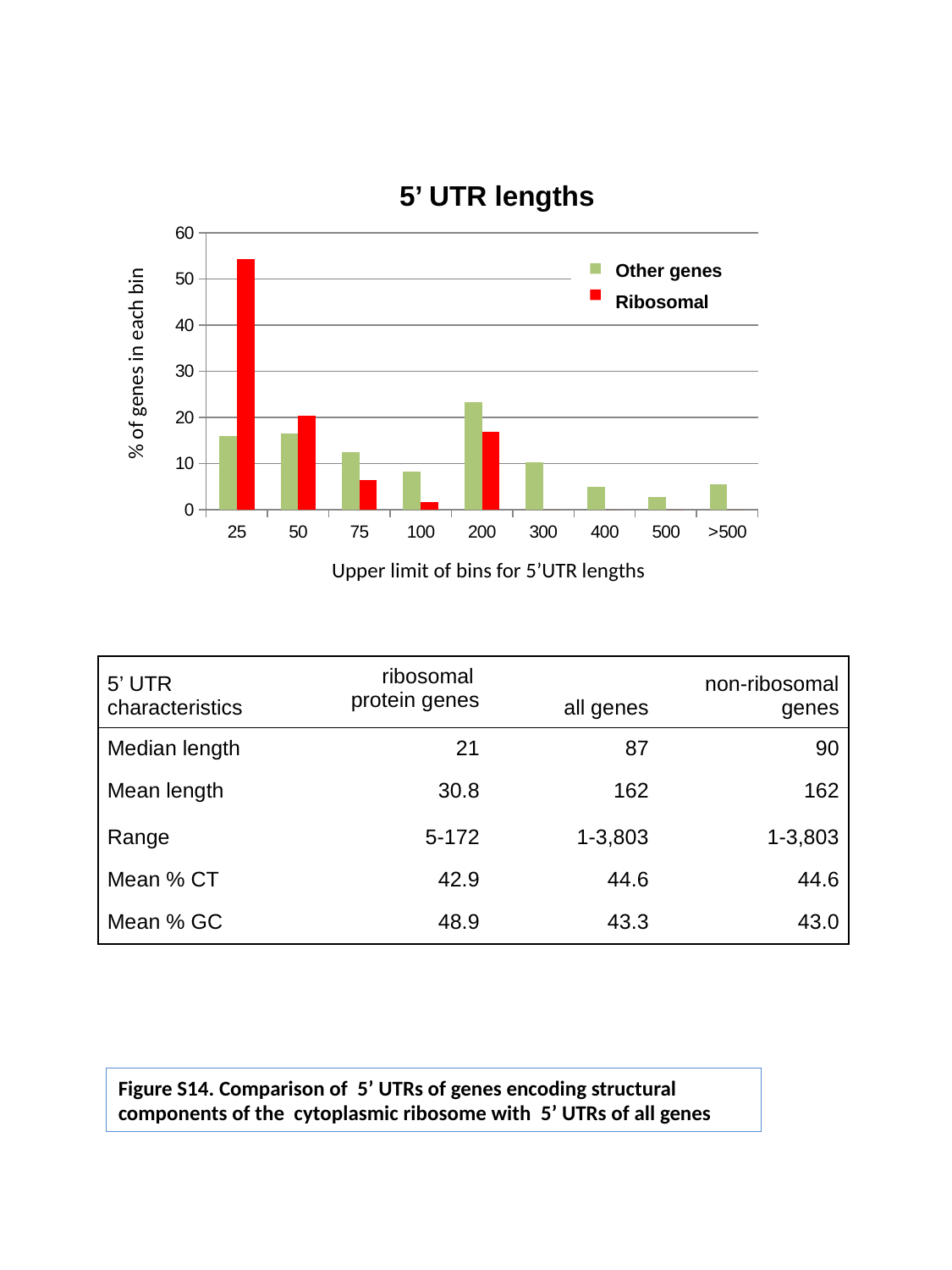

5’ UTR lengths
### Chart
| Category | Rest of the genes | Ribosomal genes |
|---|---|---|
| 25 | 15.89749968901605 | 54.38596491228071 |
| 50 | 16.50702823734295 | 20.46783625730993 |
| 75 | 12.52643363602438 | 6.432748538011696 |
| 100 | 8.197537007090434 | 1.754385964912281 |
| 200 | 23.34867520835925 | 16.95906432748538 |
| 300 | 10.2997885309118 | 0.0 |
| 400 | 5.013061326035576 | 0.0 |
| 500 | 2.74909814653564 | 0.0 |
| >500 | 5.460878218683916 | 0.0 |Other genes
Ribosomal
% of genes in each bin
Upper limit of bins for 5’UTR lengths
| 5’ UTR characteristics | ribosomal protein genes | all genes | non-ribosomal genes |
| --- | --- | --- | --- |
| Median length | 21 | 87 | 90 |
| Mean length | 30.8 | 162 | 162 |
| Range | 5-172 | 1-3,803 | 1-3,803 |
| Mean % CT | 42.9 | 44.6 | 44.6 |
| Mean % GC | 48.9 | 43.3 | 43.0 |
Figure S14. Comparison of 5’ UTRs of genes encoding structural components of the cytoplasmic ribosome with 5’ UTRs of all genes
